# How do urban green spaces influence heat-related mortality in elderly? A realist synthesis

**DOI:** 10.1186/s12889-024-17973-5

**Published:** 2024-02-13

**Authors:** Tom Cornu, Bruno Marchal, Dimitri Renmans

**Affiliations:** 1 Chair Care and the Natural Living Environment, University of Antwerp, Antwerpen, Belgium; 2 Complexity and Health unit, Department of Public Health, Institute of Tropical Medicine, Antwerp, Belgium; 3 School of Public Health, Université Libre de Bruxelles, Brussels, Belgium

**Keywords:** Climate change, Urban heat islands, Urban green space, Heat-related mortality, Realist synthesis, Social capital, Elderly

## Abstract

**Background:**

An important consequence of climate change for urban health is heat-related mortality. Vulnerable groups, especially elderly, will be the most affected. A solution put forward in many reports and policy documents is the introduction or expansion of urban green spaces. While they have a proven effect in decreasing the ambient temperature and reducing heat related mortality, the causal pathways are far from clear. Moreover, results vary for different contexts, population types and characteristics of green spaces as they are ‘complex systems thrusted into complex systems’. To our knowledge, there is no systematic synthesis of the literature that examines the mechanisms by which and the circumstances under which green spaces work to decrease heat-related mortality for elderly.

**Methods:**

We performed a realist synthesis– a theory-driven review method– to develop a complexity- and context-sensitive program theory. As a first step, a causal loop diagram was constructed which describes the possible pathways through which urban green spaces influence heat-related mortality in elderly. In a second step, one of the pathways - how they may lead to a reduction of heat-related mortality by increasing social capital - was further explored for underlying mechanisms, the context in which they work and the differentiated patterns of outcomes they generate. Literature was searched for evidence supporting or contradicting the initial programme theory, resulting in a refined theory.

**Results:**

Results show how urban green space can impact on heat-related mortality in elderly by its influence on their exposure to outdoor and indoor heat, by improving their resilience as well as by affecting their access to treatment. Urban green spaces and their interactions with social capital affect the access to health information, social support, and the capacity for effective lobbying. Several mechanisms help to explain these observed demi-regularities, among others perceived behavioural control, perceived usefulness, receptiveness, ontological security, and self-interest. If and how they are triggered depends on the characteristics of the urban green space, the population, and other contextual factors.

**Conclusion:**

Looking into the impact of urban green spaces on heat-related mortality in elderly, researchers and policy makers should take interest in the role of social capital.

**Supplementary Information:**

The online version contains supplementary material available at 10.1186/s12889-024-17973-5.

## Introduction

All over the world, urban populations are confronted by the effects of global warming. More than half of the global population is living in urban areas. It is estimated that this will increase to more than 60 per cent by 2050, the most prominent shift taking place in Africa and Asia [1, 2]. At the same time, the direct and indirect impact of climate change on human health is ever more clear [3]. Urban areas are particularly at risk of global warming due to their population density and their specificity in infrastructure, activities, and geographical distribution [4].

One of the most important direct consequences of climate change, now and even more in the future, are heat waves [4, 5]. In urban areas, these are exacerbated by the urban heat island (UHI) effect, which means temperatures are higher in densely built areas than the surrounding areas. Many factors contribute to this, including extensive sealed surface coverage, the absorption of solar radiation by building materials, limited vegetation, heat-producing anthropogenic activities, reduced air circulation and reduced nocturnal cooling [6]. Accordingly, a climate change risk assessment reports heat-related illness as the most prominent perceived health concern in more than half of the surveyed cities [7]. Symptoms range from mild effects, like fatigue, discomfort or heat cramps, to more severe effects, like heat-exhaustion, heatstroke and death [6]. Heat-related mortality is caused by a wide range of causes, of which cardiovascular, respiratory diseases and heatstroke are the most important [8].

Among the most affected people are vulnerable groups: infants, elderly, pregnant women, people with chronic conditions, and outdoor workers, but also poor, displaced and homeless people [6]. Elderly are the largest group at risk because of their pre-existing chronic conditions (respiratory disease, cardiovascular disease, diabetes, and mental illness in particular). Also the modification of physical mechanisms in the elderly, such as a reduced sweating and a reduced sense of thirst, plays a role [8]. The increase in frequency of heatwaves because of climate change led to a steady increase in the person-days of heatwave exposure for adults older than 65 and a record high of heat-related deaths in people older than 65 years in 2019 as reported by the Lancet Countdown on Health and Climate Change [7].

One of the most frequent mentioned strategies to mitigate the effects of climate change in cities is the introduction or expansion of urban green spaces (UGS) [9], which has been demonstrated to reduce heat-related mortality [10–15]. Additionally, they provide many other ecosystem services, such as recreation, air filtering, water management and carbon sequestration, which all contribute to human wellbeing [4, 7, 16, 17]. Finally, they contribute to the resilience of cities [18]. However, most studies of their impact on heat-related mortality are observational [19]. While a statistically significant correlation has been established, the causal pathways underlying these effects are far from clear. Moreover, UGS’s effects differ across contexts, population types and UGS with different characteristics [7, 10, 11, 13, 20–24]. Different pathways may explain this heterogeneity, from cooling of ambient temperature to the improvement of general health.

Further complicating the issue is the fact that UGS are “*complex systems thrusted into complex systems*” [25]. UGS can be considered as (managed) natural ecosystems, which are nested in larger systems, such as a city or a larger ecosystem. At the same time, UGS are places where people carry out activities, and thus social spaces. The interaction between the social use of UGS and the UGS-as-an-ecosystem can shape the impact of UGS on heat-related mortality. Some studies mention factors influencing the impact of UGS, while others describe possible pathways for different health aspects [10]. To our best knowledge, there is no systematic synthesis of the literature that has examined the mechanisms by which and the circumstances under which they work to decrease heat-related mortality for the elderly. Using a realist synthesis approach, we aimed at contributing to a better understanding of the relevant causal mechanisms, by identifying relevant frameworks and causal pathways that may explain how UGS influence health-related mortality in the elderly living in cities.

## Methodology

We used realist synthesis (RS) to review the existing literature on the topic. RS was developed by Ray Pawson [18] and has its roots in scientific realism and, to some extent, in Bhaskar’s critical realist philosophy [26]. Different from other review methods, RS is focused on identifying the mechanisms and contextual factors that explain how a particular intervention leads to an outcome. The context-mechanism-outcome configuration is the heuristic used by realists in the analysis. The latter identifies what works, for whom, under what circumstances and why. In this study, we define causal mechanisms as *“behavioural mechanisms, often occurring at the microlevel, although not restricted to it*” [19, p. 104]. The context should be seen as pre-existing factors which allow the intervention to trigger the mechanism. This includes programme inputs (resources, rules, and components), the characteristics of participants and the institutional, cultural and historical surroundings [27].

A RS starts from an initial programme theory (IPT), which is informed by previous research, substantive theories, grey literature, expert knowledge or by personal hunches. The reviewers then search for evidence to support, reject of modify the IPT, resulting in a refined programme theory [25]. In this study, we followed the steps and procedures proposed by Pawson and colleagues as well as the Rameses guidelines [18, 25].

### Define the scope of the review

We started with a literature review looking for frameworks, programme theories and contextual factors explaining the impact of urban green spaces on heat-related mortality in elderly. We focused on reports from international and supranational institutions (incl. WHO, World bank, IPCC and the European Commission), and relevant overview papers. We summarised the findings in a ‘rough’ causal loop diagram^1^. Causal loop diagrams (CLD) enable us to visualize complex systems. The arrows represent causal linkages between different elements of the system. They are labelled positive or negative meaning that a change in the first variable leads to a change in the second variable in the same direction or in the opposite direction respectively. Interactions between elements lead to our outcomes of interest [27]. We used the diagram to integrate and differentiate several IPTs. In the Results section, we describe how we developed the CLD and how we chose IPT 5, focusing on the influence of the social dimension of UGS. to be checked and refined in the following steps.

### Search for and appraise evidence

In a next step, we searched for evidence to support, reject or modify (parts of) the IPT. We carried out a purposive sampling of information-rich articles from the list of papers identified by our literature review. We also used the snowballing technique (citation and reference tracking) based on these articles. To pursue some potential leads that surfaced when we further refined the IPT, we performed additional searches on Scopus, Embase, PubMed, Web of Science and Google Scholar. All evidence was appraised on relevance.

### Extract and synthetise findings

Data were extracted using NVivo software. We coded the text using a coding frame that was based on the IPT. Child nodes were used when we found new variables within a certain causal relation. We remained open for explanations not covered by the IPT.

In a next step, for the most relevant and information-rich causal relations, the linked data were analysed for plausible CMO-configurations (CMOC). If not enough evidence was available to accept or reject them, additional literature searches were performed until saturation was reached. The evidence for each CMOC was summarized and this will be presented in the Results section. We kept a detailed account of the analysis in order to maintain transparency [28].

### Draw conclusions and make recommendations

We modified the IPT on the basis of the CMOCs we identified. We highlighted the most important findings and translated these into policy recommendations.

## Results

### Defining the scope of the review

#### Using a causal loop diagram as a way to integrate the findings of the review

**Fig. 1:**
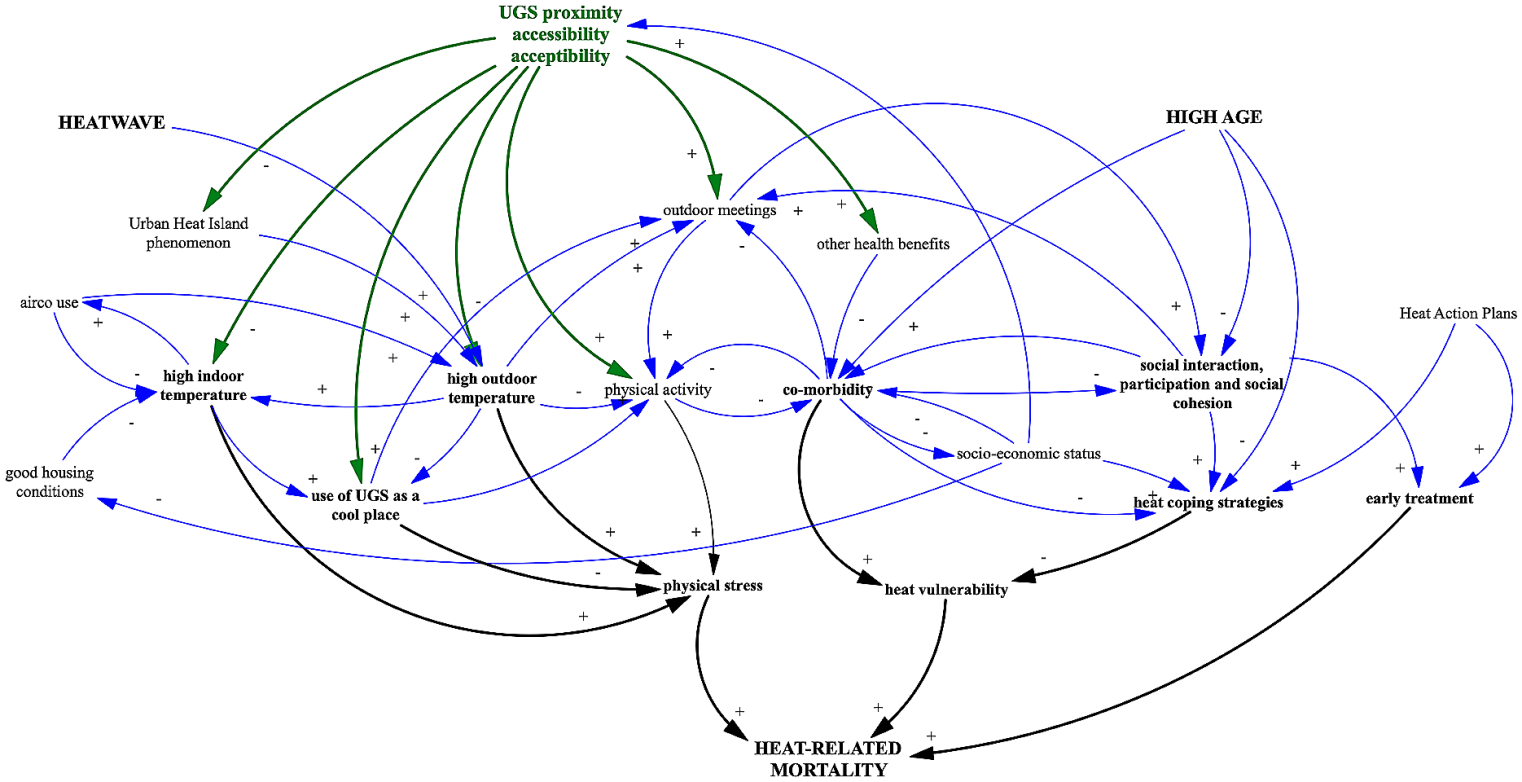
Causal loop diagram of relationship between UGS and heat-related mortality

As explained above, we developed a CLD to capture the relationships between UGS and heat-related mortality and to show the links between the pathways (Fig. 1). It was constructed on the basis of three models: a pathway from heat exposure to heat-related mortality [5], a set of factors influencing thermoregulation [8] and a causal model of the impact of UGS on health and well-being [23]. We then added additional information found in reports of international agencies and overview articles on UGS and heat-related mortality [4, 6, 8, 10, 16, 17, 19].

The CLD shows how heat-related mortality in elderly is the result of a vulnerability to heat, exposure to heat (heat stress) and the (lack of) access to (early) treatment [5, 8].

- Heat vulnerability is determined by the individual’s heat sensitivity and the coping strategies used. Heat tolerance diminishes with age due to natural patterns of senescence and a higher prevalence of chronic conditions or co-morbidity [6, 8, 17]. Additionally, high age will have a negative influence on the use of heat-coping strategies because of this co-morbidity but also because of social isolation [29].
- Heat stress can be caused by high outdoor and indoor temperatures and is aggravated by physical activity [6, 8, 10, 16]. Co-exposure to air pollution can aggravate physical stress and heat-related mortality [30].
- Access to early treatment depends on a number factors (geographical, financial and other barriers), social isolation being one of them [18].

UGS can affect all the pathways contributing to heat-related mortality. First, UGS in proximity can lead to a reduction of the outdoor and indoor temperature by reducing the urban heat island effect and creating shading [10, 24]. Large parks and areas of woodland can function as a cool place for elderly to go to in times of heat, reducing their heat stress [4, 8]. Second, they can decrease co-morbidity by reducing exposure to environmental stressors (e.g. heat, air pollution), building and restoring capacities (e.g. by enhancing physical activity, positive effects on cognitive functioning) [11, 23, 31–34]. Third, urban green spaces that are nearby, accessible and acceptable contribute to outdoor meetings, which increase social interaction, participation and cohesion [11, 16, 19].

Since the ‘cooling pathway’ can be split in three sub-pathways, we discern a total of five IPTs (Table 1). A short overview of the IPTs, including related evidence, can be found in annex A.

**Table 1:** Initial programme theories of how UGS influence heat-related mortality in elderly persons

**Reduction of exposure**
IPT 1: By reducing outdoor temperature UGS reduce heat related mortality for elderly
IPT 2: By reducing indoor temperature UGS reduce heat related mortality for elderly
IPT 3: By providing a cool place UGS reduce heat related mortality for elderly
**Reduction of vulnerability**
IPT 4: By reducing co-morbidity UGS reduce heat-related mortality in elderly
**Increase in heat-responsiveness**
IPT 5: By increasing social interaction, participation, and cohesion UGS increase the heat responsiveness of elderly

#### Choosing a programme theory

In this paper, we focus on how we refined IPT 5, which we chose because the influence of the social dimension of UGS on heat-related mortality is less obvious and less studied than the other pathways [6, 35, 36]. While developing the CLD, it became clear how this IPT is intertwined with the other pathways. It can, for instance, influence how people perceive their surroundings, whether they perform physical activities, whether they visit cool places and how they cope with high indoor and outdoor temperatures.

##### Social capital as a central concept

We elaborated IPT 5 drawing on the theory of social capital, a concept that is broadly used to describe the impact of the social dimension on health [37–42]. It provides a theoretical foundation for the influence of the social dimension of UGS on health and well-being pointing to mechanisms and social features, such as norms, trust, reciprocity and strong networks, that can lead to access to resources and social support, and to other social processes, such as participation and social influence [42].

Social capital can be viewed as an individual or a collective asset. The former approach, of which Bourdieu is the most prominent proponent, focuses on the ability of people to obtain personal benefits from their membership of social networks and other social structures. The latter, based on the work of Putnam, stresses how social capital can also have the characteristics of a common good, benefiting people with poor social connections because of spill over effects [42].

Different definitions are used. The most mentioned in the field of public health is that of Putnam (1995) as cited by Norstrand & Xu (2012): “*features of social organization such as networks, norms, and social trust that facilitate coordination and cooperation for mutual benefit*” (p. 326). Portes (2009) differentiates between the sources and effects of social capital, the former consisting of the characteristics of social networks (motivations to make resources available such as internalized norms, solidarity and common fate) and the latter of the resources provided (information, social support and opportunities) [42]. Additionally, the differentiation between bonding, bridging, and linking social capital seemed interesting to our analysis. ‘Bonding’ concerns the strong ties within a network that strengthen common identities and that functions as a source of help and support among members. ‘Bridging’ relates to the weaker ties linking people from different networks, which can provide access to important sources of information and resources, while ‘linking’ refers to the vertical ties with formal and institutionalized power hierarchies [42].

##### Construction of the CLD for IPT 5

**Fig. 2:**
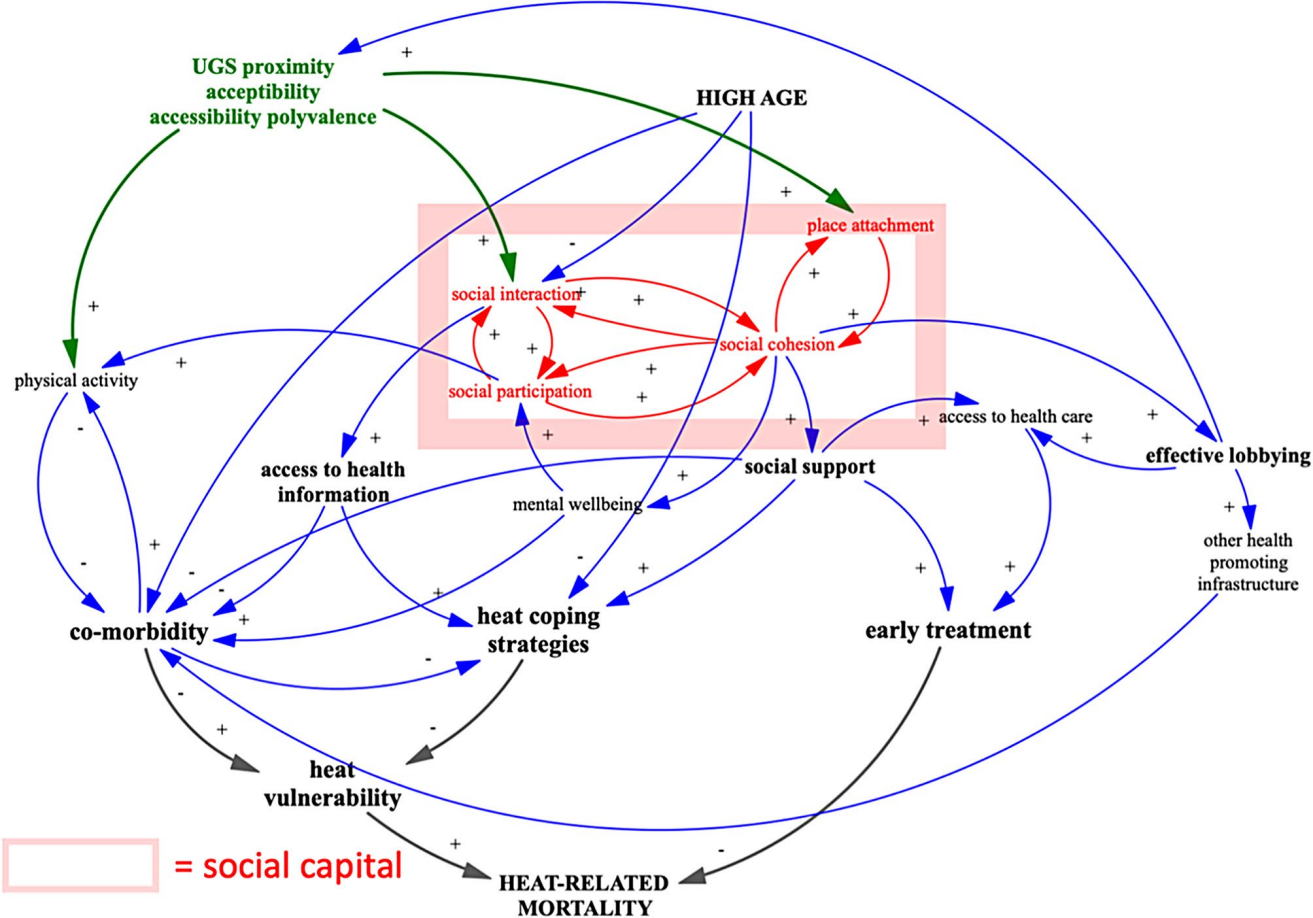
Causal loop diagram for IPT 5 social capital

In a next step, we integrated social capital in the CLD (Fig. 2). It is shown as a rectangle encompassing the components relevant for our research question: social interactions, social participation, social cohesion and place attachment. Placed at the centre, it indicates two important phases: UGS influencing social capital and social capital influencing heat-related mortality.

The presence of UGS in a neighbourhood can enhance social interaction, social participation and social cohesion. Likewise, it has been shown that UGS stimulate place attachment. Both influences will be determined by characteristics of the UGS, including proximity, accessibility, acceptability, and polyvalence [43]. For reasons of readability, we have merged these four characteristics into one condition in the CLD.

The resulting increase in social capital may influence the health status of people and more specifically affect heat-related mortality: it contributes to increased access to health information, to informal health care and support in case of sickness and to effective lobbying to obtain potentially health-promoting public goods [44]. In this way, increased social capital can reduce co-morbidity, stimulate the use of heat-coping strategies, and increase early treatment, leading to reduced heat-related mortality.

### Searching for and appraising the evidence

Combining the papers from our first scoping review with papers identified through a specific search on the basis of IPT 5, we identified 45 relevant articles. Table S1 in annex B presents the papers in detail (incl. the study design, geographical scope and peer review status).

### Extraction and synthesis of the findings

In our analysis, we identified 8 CMOCs. Four relate to the relationship between UGS and social capital. Four others show how social capital can influence social support, and lead to heat-coping strategies and effective lobbying.

Below, we present a summary of the evidence for each CMOC, indicating how specific conditions can trigger or modify mechanisms leading to outcomes. For the description of the CMOCs we use the if-then-because structure where ‘if’ represents the context, ‘then’ the outcome and ‘because’ the mechanism [45]. Annex C gives a more extensive overview of the evidence related to each CMOC.

#### From urban green spaces to social capital

We first look at how UGS contribute to social capital, with the intermediate step of the actual use of green spaces. CMOCs 1 and 2 represent the relation between UGS and use, CMOC 3 that between use and social interaction, and CMOC 4 between use and place attachment.

##### CMOC 1. Capability

***If****UGS are available in the proximity of their home and****if****they are perceived as accessible and safe and as a place where they feel at ease*, ***then****elderly people will use urban green spaces****because****of high perceived behavioural control.*

For elderly persons to visit green spaces, it is important they feel capable of dealing with objective and subjective barriers. Perceived behavioural control is defined as “*an individual’s perceived ability and ease to perform certain behaviours*” [46, p. 3]. The literature indicates a wide range of contextual conditions that have an influence on perceived behavioural control of elderly people.

First, it is important that elderly persons believe UGS are accessible. Proximity is important as declining health makes elderly people more dependent on their direct living environment [47–50]. Decreased mobility increases the need for safe walking infrastructure on access routes as well as in the parks [51, 52]. Likewise, the absence of supportive facilities like benches or toilets can be seen as a barrier [51, 53]. Specifically during periods of heat, shading on access routes as well as in the UGS will be important [47, 54, 55].

Second, perceived safety influences whether elderly feel capable of visiting UGS. People who fear being assaulted limit their outdoor activities [56]. This is even more so for elderly persons because of weak physical capability and decreasing self-efficacy [48]. Elderly women can feel more vulnerable to anti-social behaviour, like harassment by drunk men [51]. Studies show that perceived safety of green spaces is related to a person’s satisfaction with his or her social network [57], the larger socioeconomic environment of a community and the level of maintenance [48, 52]. As active use is an important factor for security, Peters et al. [58] warn for over-regulating design and space.

Third, elderly visit green spaces in which they feel at ease. Older people mostly visit parks with others, especially with their partner and to meet people they know [53, 58]. Isolated elderly persons could feel less inclined to use green spaces. Perceived differences with other visitors, including negative perceptions of other people using the green spaces (e.g. youth) can lead to self-exclusion [59, 60]. Elderly in the study of Menec et al. [52] describe their need for a culture of respect. Other barriers mentioned are crowding and the presence of dogs [47].

As deprived neighbourhoods tend to score badly on all of three above-mentioned characteristics, elderly residents can feel restrained in using green spaces in such neighbourhoods [48, 61].

##### CMOC 2. Usefulness

***If**** elderly people perceive urban green spaces to address their needs, such as being a place of comfort or a meeting place*, ***then****they will visit them****because****of their perceived usefulness.*

Elderly persons will visit a green space if they think it will fulfil one of their needs. They look for specific attributes of UGS in function of their individual and contextual needs, and their cultural preferences [58]. During heat periods, elderly people living in a hectic, polluted city will look for a place of comfort with lower levels of noise, air pollution and heat [51]. For elderly living alone, the social function of UGS can be important [62]. They can see UGS as a place to meet people and where people come together across generations [52, 61]. During the COVID-19 pandemic, UGS were used as a safe meeting place [55]. Furthermore, when having grandchildren or a dog, elderly people use UGS for its recreational function [51, 60]. Contrary to UGS being promoted for their potential of increasing physical activity, this is not a main reason for elderly persons to visit them. It is found that physical activity is better considered as a by-product as elderly need to walk from their house to the UGS [51, 61].

Elderly people assess the usefulness of UGS on the basis of their structure, infrastructure and design [58], and this will be interpreted through a subjective lens [59]. Studies have shown how urban vegetation, for instance, has a positive impact on people’s perception of heat that goes beyond its objective heat reduction [63]. How elderly people consider attributes of usefulness of UGS is also based on perceived ‘ambiences’, based on the functions and ‘image’ of the UGS. Respondents in the study of Peters et al. [58] characterized one park with expressions like ‘seeing other people and being seen’, another was seen as fit for everyday use. An important factor in these interpretations is place attachment, which can influence experiences and lead to a positive perception of UGS [63, 64]. Li et al. [65] describe how in their study place attachment fully mediated the association between UGS components and actual use.

##### CMOC 3. Social interaction

***If**** elderly people, especially socially isolated persons, use green spaces where they feel comfortable, or where they meet other people, they feel connected to or accepted by*, ***and if****the UGS has a supportive design, facilities and activities*, ***then****they will have more social interaction****because****of increased receptiveness for social interaction.*

Studies have shown a positive relation between the presence of public green space and social ties [49]. UGS may indeed promote social interaction through mechanisms such as stimulating receptiveness for social interaction.

First, receptiveness for social interaction will increase if people feel comfortable and relaxed. UGS provide shadow, privacy and sound buffering from surrounding environments and can have restorative effects from stress [49]. Studies have shown how the availability of greenness along with safety and maintenance influences social interaction [66]. In the same way, establishing contacts is mentioned to be more difficult if the characteristics of the neighbourhood are not conducive [67].

Second, when elderly people see UGS as a meeting place, they may enter it with a mindset for social interaction [48, 53, 64]. Schmidt et al. [61] mention how social relationships or casual encounters around seating places with different neighbours were an important reason for the residents to visit UGS. This can be especially important when people do not have many other contacts. Kemperman and Timmermans [48] mention how social contacts are more likely to happen in a neighbourhood if residents have few alternatives.

Third, elderly can be more receptive to interaction in UGS when they perceive it as a place where they belong and are accepted by the other visitors. Important factors for this were already mentioned in CMOC 1. Peters et al. [58] describe how parks can achieve as ‘everyday places’, in which people feel at home, or as a ‘world of strangers’, which are open and accessible spaces that attract a variety of people.

Finally, certain characteristics of and activities in UGS can enhance openness to social interaction, social gathering and shared experiences. For example, benches, playgrounds where elderly go with their grandchildren, and community gardens can promote interaction and the development of friendships [48, 51]. Shared experiences will be especially important for social interaction with strangers and thus contribute to produce bridging social capital. While social interaction of elderly people in UGS will be mostly with people they know, an external stimulus (e.g. children, dogs, balls) can provide an opportunity for contact between strangers, a process called triangulation [58].

##### CMOC 4. Feeling at home

***If**** elderly persons use UGS with physical features they think are important, with people they feel connected with and where they are at ease with and this for a longer period and with a certain frequency*, ***then****they will develop place attachment****because****of feelings of ontological security.*

Our review points to physical features and conditions of UGS that make it easier for elderly to become attached to the green space (and to their neighbourhood on general). Such features stimulate ‘feeling at home’, which is captured by the concept of ‘ontological security’. The latter is associated with the need of human beings for continuity of their self-identity and a constancy in their surroundings. To feel more secure in a chaotic world that challenges their identity, people seek for familiar places which are transformed into ‘known’ and ‘own’ places [68].

First, physical features of UGS which people like or think are important for them, will enhance the feeling of being at their ‘own’ place. Consequently, participation in the design of UGS can foster feelings of belonging and identification [53]. Naturalness of UGS may be another reason for attachment, as studies have shown that humans have an innate predisposition to affiliate with natural places [49, 62].

Second, when people visit a park where they know other people a sense of familiarity is stimulated [58]. When people emotionally connect with each other and have attention to the space they use and share, this can translate into an affinity for this shared environment [65, 69]. However, different groups and individuals may struggle over the production, occupation and control of place [68]. Consequently, socio-cultural groups can have a different degree of UGS attachment [58]. Linked to this, for elderly people to have a sense of familiarity, it is important they understand which activities are appropriate and which not. The design and organisation of green spaces can give direction, but also a shared social background and social norms are important [68], potentially making it harder for minority groups to feel at home.

Third, a precondition of feeling secure is feeling safe. Studies show how UGS that are perceived as safe enhance attachment [57]. Conditions influencing (perceived) safety were mentioned in CMOC 1.

Finally, feeling familiar with a place can be linked with length and frequency of contact. The longer people live in a neighbourhood, the more they visit an UGS, the more they become attached. Frequency of visits is positively related to green space attachment [62, 68]. For instance, women who lived longer at a certain address having a higher sense of belonging [56].

#### From social capital to heat-related mortality

CMOC 1–4 presents the conditions and mechanisms that can contribute to positive social capital. CMOC 5–8 indicates how this can play a part in the reduction of heat-related mortality by increasing social support, the use of heat-coping strategies and effective lobbying.

##### CMOC 5. Selfish support

***If****elderly persons belong to (part of) a neighbourhood community with high social capital (generating trust in reciprocity) and****if****their community members perceive a need for help*, ***then****the elderly will receive social support from their community****because****of the self-interest of community members (especially when having more distant relationships).*

Putnam describes how in a high social capital community, people cooperate on the basis of expected reciprocity, which is encouraged by high levels of interpersonal and generalised trust [42]. Correspondingly, studies show how social support is linked to trust in reciprocity, pointing to the mechanism of self-interest.

Reciprocity is a driving force for social support and in a lot of cases a necessary condition. Brewster et al. [66] found that norms of trust and reciprocity foster social network ties that lead to neighbour-to-neighbour assistance. Likewise, Neufeld & Harrison’s work [70] on reciprocity and social support in caregivers’ relationships showed how relationships were mainly based on some kind of reciprocity. However, there was a difference in expectations on return in their ‘give and take’- relations. In close relations, caregivers can be content even with a ‘constructed reciprocity’^2^, while in more distant relationships, the absence of reciprocity will lead to the end of the relation [70].

This conditionality can influence who will receive what kind of social support and under what conditions. More distal relations– with friends and neighbours - are increasingly recognised as a source of social support for elderly [48, 71], especially for persons isolated through poor health, limited mobility, financial constraints or a lack of access to transport [56]. In such cases, a more selfish form of social support will occur for these elderly. Likewise, some research undermines the romantic vision of neighbourly support: social fragmentation can make social capital and social support only accessible for the selected members of subgroups [42].

Whether social support will be given will also depend on perception of need. Studying social support during heatwaves, Wolf et al. [70] found that many elderly people perceived dealing with heat as ‘common sense’ and that a high value of individual independence would stop people from interfering. When other respondents did interfere, this was based on previous experiences or serious illness and disability of the care-recipient.

##### CMOC 6. Selfless support

***If****elderly people live in a neighbourhood with positive social capital, which promotes ‘helping out norms’ and****if****there are enough resources*, ***then****they will receive social support****because****of motivation of community members by internalisation of these norms.*

People do not only give social support out of self-interest. They may also be willing to make resources available because of internalized social norms and because of solidarity with people with whom they identify as sharing a common fate [42]. Self-determination theory explains how social norms can motivate people to act in a certain way, not only because of rewards or sanctions, but because of internalization of these norms [72].^3^

A study on the determinants of informal care-giving points out the importance of norms and beliefs for helping out neighbours and friends [71]. Likewise, research on informal family care points to how social norms could be a possible explanation for geographical differences in informal care giving [73, 74]. Moreover, Uehara [75] describes the role of moral norms like altruism and needs-based redistributive justice, especially in the social support for elderly. She shows how a reciprocity norm - the obligation felt to reciprocate help– can play an important role. The study of Neufeld & Harrison [70] supports the co-existence of reciprocity and norms, but their study found that social support based on norms without reciprocity can lead to negative feelings in the caregiver [70].

##### CMOC 7. Heat-coping

***If****elderly persons live in a neighbourhood with positive social capital (where they receive social support)*, ***if****they are mentally and physically healthy, and literate and****if****they have access to good health information and other necessary resources*, ***then****they will use the right heat coping strategies****because****their self-efficacy is high enough.*

Prevention strategies are important in reducing heat-related mortality in elderly as treatment often comes too late [76]. Bandura defined self-efficacy “a person’s belief in his or her ability to succeed in specific situations or accomplish a task” [77].

First, social capital in a community enhances self-efficacy in elderly by facilitating the diffusion of information. Social capital, characterized by trust, has been shown to facilitate faster and wider diffusion of (health) information and knowledge [42, 64]. This diffusion can happen through informal discussions [69], but also by observing and learning from each other’s behaviour [78], whereby trusted peers function as role models [42]. Second, in addition to information diffusion, social capital can contribute to elderly’s self-efficacy. Social support, and even the belief in the presence of social support, can strengthen self-esteem and self-efficacy [69, 79].

However, for social capital to effectively increase the use of the right heat-coping strategies, elderly people need to be aware that they are at risk, require correct health information and need to have access to the necessary resources. Research on collective efficacy during natural hazards shows how social cohesion can lead to low risk perception [79]. Moreover, the study of Wolf et al. [70] showed how elderly people who are part of a supportive network did not perceive themselves at risk from or vulnerable to the effects of extreme heat. They point out how bonding networks, due to a knowledge deficit and the importance they attach to individual independency, could increase heat vulnerability, and prevent the use of heat-coping strategies. Furthermore, Norstrand & Xu [80] describe how communities cannot benefit from social capital for health when lacking the necessary resources.

Additionally, individual characteristics shape self-efficacy. A low education level and low health literacy are correlated with a poor uptake of preventive services. Moreover, the decrease in cognitive processing in elderly people may explain a lower perception of risk to health and lower adoption of protective behaviour [81]. Likewise, disability and low income were found to restrict elderly people in taking actions to reduce their heat vulnerability [82].

The above-mentioned characteristics that contribute to vulnerability– inadequate information, lower risk perception, lack of resources and complicating individual characteristics ─ point to the added value of bridging and linking social capital [83], as well as of health programmes, [84] where both can increase access to the right health information and resources.

##### CMOC 8. Collective lobbying

***If****elderly persons are living in a neighbourhood with high social capital*, ***if****they share goals*, ***if****they are effectively engaged in participation processes and****if****the community has access to enough resources*, ***then****elderly people can effectively lobby for health-related services and infrastructure*, ***because****of collective efficacy.*

Research shows how social capital can increase the ability of a community to influence the provision of community services [42, 66]. Evidence in literature describes different aspects of social capital that point to the mechanism of collective efficacy or ‘a group’s shared belief in its conjoint capabilities to organise and execute the courses of action required to produce given levels of attainment’ [64, p. 477].

For communities to believe they can reach a common goal, a common purpose is a prerequisite. While communities with high social capital by definition are in a good position to achieve collective action, the stronger and more concrete their purpose, the more successful it can be. VanHoose & Savini [85] describe a common goal contributes to common identity and the maintenance of group integrity, essential when negotiating with formal institutions.

Both bonding and bridging social capital contribute to the belief in conjoint capabilities. The former increases internal capacity building and provides access to internal resources, while the latter helps to obtain resources unavailable within the group via intermediaries and experts [85]. According to Eriksson [42], certain community members, such as strong leaders, have an important role to play. In her study of a community that successfully opposed a political decision to close a primary health care centre, Eriksson also points to other characteristics of social capital that help people to belief in their joint capability [42]. She found that the community was characterized by a history of high levels of civic engagement (“we have realized things before”), dense associations were important at getting people involved (“everybody is in””) and social norms obliged people to engage in their community (“everybody will do their best”).

Finally, we need to point to the danger of exclusion of elderly persons in participation processes such as community action. Enssle & Kabish mention lower participation among older people [53]. Moreover, a study by Low on procedural justice for public spaces [86] shows how minority groups within older people, such as migrants, were found reluctant to engage in participation processes. Linked to this, Van Hoose & Savini [85] conclude in their study that the need for a strong group identity carries the risk of exclusion. This may be especially the case for elderly people, in essence a more vulnerable group.

## Discussion

**Fig. 3:**
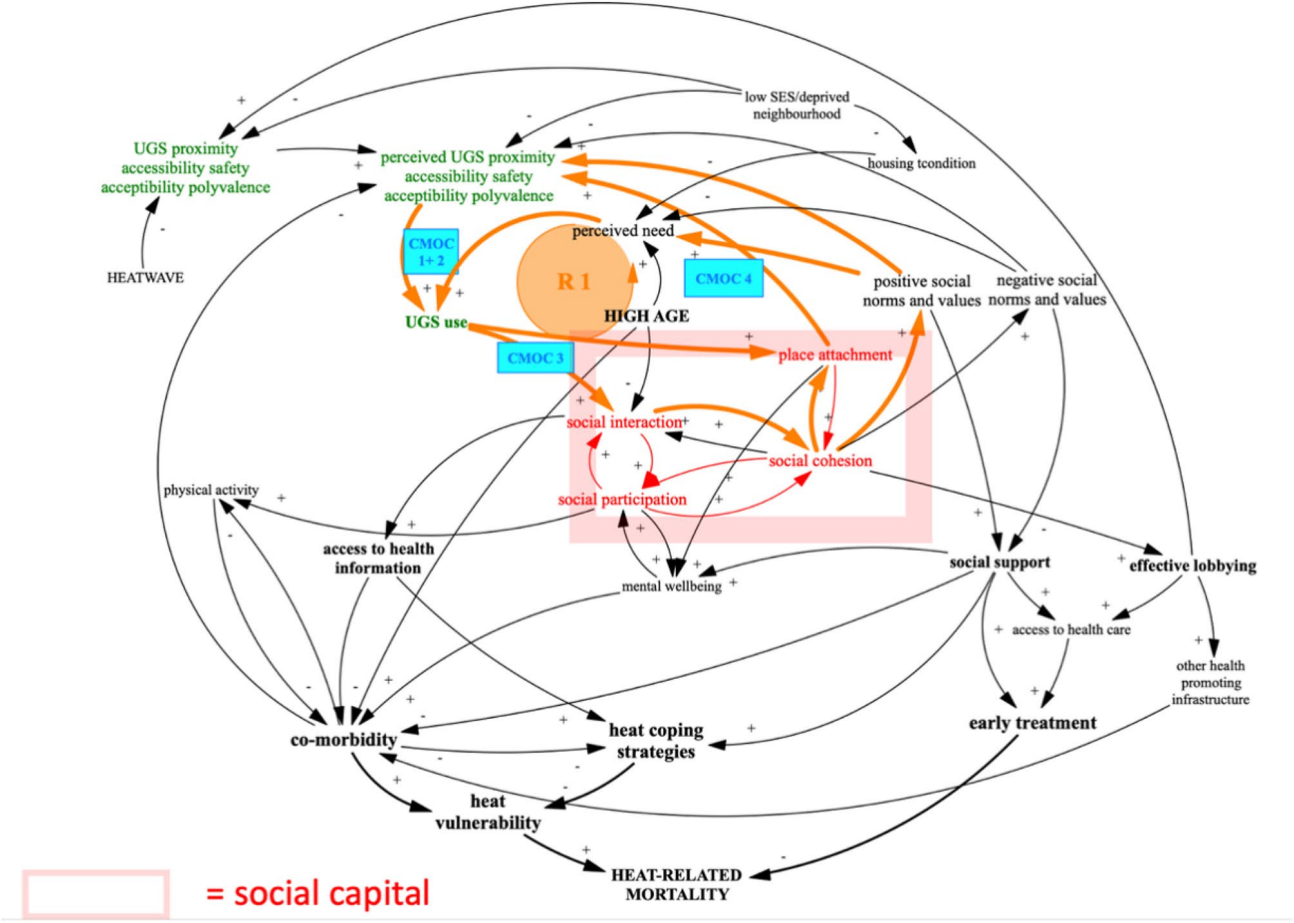
CLD for the refined IPT 5 CMOC 1–4

In this realist synthesis, we first analysed how, why and under what conditions UGS could lead to an increase in social capital. We found that a combination of individual characteristics of elderly people and features of UGS (like design, infrastructure and facilities) influence whether elderly persons feel capable of using an UGS. It will also shape how elderly people consider that green spaces address their personal needs, and how green spaces make them more receptive for social contact. Finally, it influences how they feel attached to the UGS and their neighbourhood (Fig. 3).

Our findings highlight how different aspects of social capital can influence the use of UGS by elderly people. Thus, social capital is an outcome of CMOCs 1–4 and a context factor for all of them, creating reinforcing loop R1 in our CLD. Importantly, UGS may contribute to place attachment which can lead to a positive perception of the UGS and the neighbourhood. For example, if there is vandalism in a park, elderly may visit less because of reduced perceived safety, interact less with people from the neighbourhood, which may result in less social cohesion and place attachment, possibly leading to a more negative perception of features and attributes of the park as well as of the other visitors.

Another finding is the importance of polyvalence. Green spaces that can be used in different ways may increase their use by different groups. This can facilitate the interaction between these groups by processes of triangulation, leading to more inclusive social cohesion and place attachment, and thus facilitate the creation of bridging social capital. However, polyvalence may lead to the opposite, when elderly are confronted with people they perceive as too different or as threatening.

**Fig. 4:**
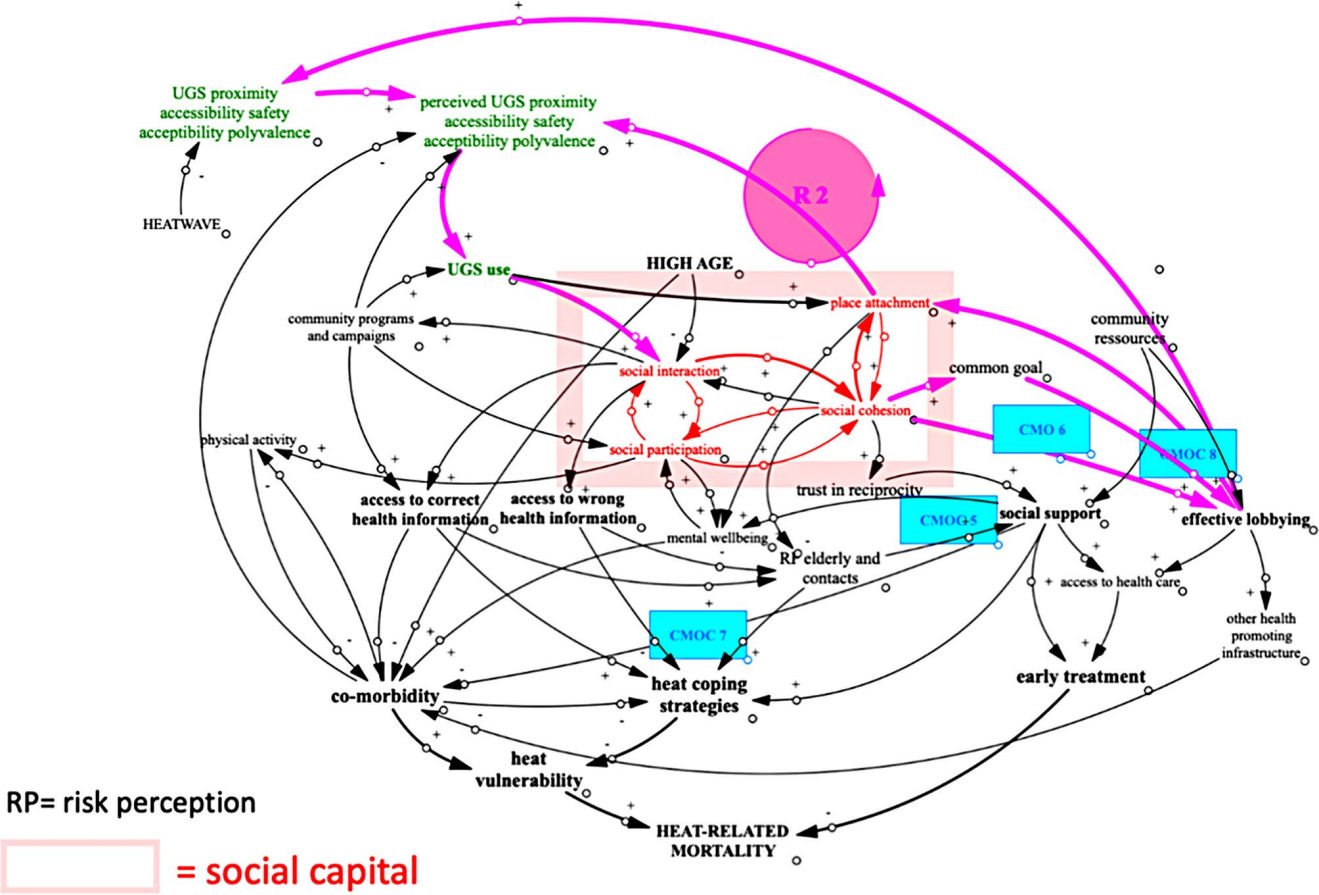
CLD for the refined IPT 5 CMOC 5–8

Second, we analysed how social capital can support mechanisms underlying the pathways leading to a reduction of heat-related mortality (Fig. 4). In relation to social support, social norms were found to play a role, but for more distal relations, our research points to the importance of reciprocity nested in self-interest. Social support can also be restricted to members of the same subgroup. Both the focus on self-interest and on subgroups could suggest a less romantic vision of the ‘supportive community’: low socio-economic status groups may have less access to social support as bridging to higher socio-economic status groups - who have access to more resources - will be difficult. Likewise, Eriksson [42] mentions how people with higher education are shown to have more access to bridging social capital.

Additionally, social capital can strengthen elderly’s capability of using the right heat-coping strategies as it can facilitate the diffusion of health information and can improve their self-esteem and self-efficacy. However, our research points to the danger of depending solely on bonding social capital as it can decrease risk perception and possibly give access to the wrong health information. Bridging social capital and health programs can counteract this by giving access to alternative information sources.

Under certain conditions, social capital can contribute to collective lobbying. Our research highlights the importance of group identity and having a concrete common goal, while pointing to the danger of exclusion of elderly in participation processes. Collective lobbying can lead to a second reinforcing loop R 2 in our CLD as we mentioned how social capital can be enhanced by the presence of physical features people think are important.

Although we only focused on one IPT, the scope of our research remains relatively broad. We acknowledge that our review only serves as a first step in constructing a theory explaining the impact of the social dimension of UGS on heat-related mortality. It needs to be refined by further research. Nevertheless, the results are important for researchers as well as for policy makers. For the former, they are a starting point and an invitation to further refine the theory by adding their research or knowledge to one or more of the topics addressed. More specifically, further clarification of the pathways by which social capital influences health and how primary health care can intervene in this, is useful as it can help to find ways of strengthening urban resilience.For the latter, our findings may serve as ‘points of attention’ when planning UGS as an adaptation measure for heatwaves (summarized in Table 2).

As realist methodology aims at explaining how mechanisms are triggered or not under certain circumstances leading to different outcomes, the CMOCs in this article are relevant in different geographical and socio-economic contexts While they’re not exhaustive in contexts and outcomes, they point to mechanisms that play an important role in all of these contexts.”

Furthermore, reflecting on the process of our analysis, we noticed that, while combining realist synthesis with the causal loop diagram tool helped our analysis, the need to give a sign to the causal arrows felt in conflict with realist theory, as context could influence and possibly reverse this sign. Even if this may lead to making the CLD less readable, splitting up variables and analysing processes in more detail may be necessary.

**Table 2:** Points of attention for policy makers

When thinking about UGS to address UHI, also think about their social function as it is intertwined with all pathways to health
To support the contribution of UGS to building social capital, next to a supporting design, infrastructure and facilities, measures focusing on social cohesion and place attachment, of ‘feeling at home’ are important: they reinforce the potential by increasing a positive perception of the neighborhood
For UGS to lead to social support, a better use of heat-coping strategies and effective lobbying for health-promoting measures for ALL elderly, attention should be paid to an inclusive social cohesion as well as the availability of the right health information and other resources • Polyvalence and measures that increase triangulation may contribute to this if one can ensure that different groups feel safe and at ease with each other • Information campaigns and other (heat) health programs may contribute to a correct risk perception and the right heat-coping strategies • Participation of elderly in the design may improve not only accessibility and but also acceptability and ‘feeling at home’

## Conclusion

Heat-related mortality in urban elderly is increasingly becoming a problem because of climate change. Urban green spaces are often proposed as a solution. Most research focuses on the cooling capacity of greens paces as an explanatory pathway for its impact on health. Using a realist synthesis, we found that the social dimension of urban green spaces is important. While policy documents on heat-related mortality mention social isolation as a risk factor, our research, drawing on the theory of social capital, stresses how the impact of the social dimension is much wider and, indeed, how social mechanisms can affect the different pathways leading to a reduction of heat-related mortality.

### Electronic supplementary material

Below is the link to the electronic supplementary material.

**Supplementary Material 1: Annex A:** Explanation on IPT’s

**Supplementary Material 2: Annex B:** List of articles with evidence for CMOC’s

**Supplementary Material 3: Annex C:** Selected evidence in literature for CMOC’s

## Data Availability

All data generated or analysed during this study are included in this published article [and its supplementary information files].

## Footnotes

1 ‘Rough’ in the sense that it was constructed to serve as a conceptual framework and that, in this stage no loops were identified yet.

2 ‘Constructed’ means they labelled observations of behaviour and responses (of a care-recipient unable to communicate) as supportive actions.

3 According to self-determination theory, internalization of norms occurs at different levels, whereby norms shaped the type of motivation over a continuum from external to internal motivation.

## Abbreviations

UHI: urban heat island
UGS: urban green space(s)
RS: realist synthesis
IPT: initial programme theory
CLD: causal loop diagram
