# Supplementary material for: How do urban green spaces influence heat-related mortality in elderly? A realist synthesis
Source: BMC Public Health. 2024 Feb 13;24:457. doi: 10.1186/s12889-024-17973-5 (PMC10865713; doi:10.1186/s12889-024-17973-5)
Supplement: Supplementary file 1 — Supplementary Material 1: Annex A: Explanation on IPT’s [file 12889_2024_17973_MOESM1_ESM.docx]

## Annex A: some explanation on IPT’s

**Cooling**

- IPT 1: By reducing outdoor temperature UGS reduce heat related mortality for elderly

UGS can reduce peak daily and night temperature by their effect on UHI’s, this by cooling through evaporation and shading as well as the reduction of impervious and heat storing materials (1). As an increase in ambient temperature is proven to be correlated to heat-related mortality, the counter-balancing effect of UGS will reduce heat-related mortality. Contextual factors mentioned to influence this effect are regional climate and urban geometry, size, proximity of the UGS and type of vegetation (1–3)

- IPT 2: By reducing indoor temperature UGS reduce heat related mortality for elderly

As people in general, and elderly in particular, stay a lot indoor, especially the indoor temperature will be important (4). UGS can reduce the indoor temperature indirectly by cooling of the ambient temperature or directly by shading. As UGS can reduce the UHI effect and thus night-time temperatures, they can reduce indoor temperature by improving night-time cooling (2).

- IPT 3: By providing a cool place UGS reduce heat related mortality for elderly

For heat-related mortality and morbidity, the duration of the heat stress is important. Spending a few hours in a cool place can be protective. One of the measures mentioned in Heat Action Plans is the provision of cool places during a heatwave (4). Especially large parcs and areas of woodland will have a lower temperature (5) and thus are able to function as a cool places.

**Co-morbidity**

- IPT 4: By reducing co-morbidity UGS reduce heat-related mortality in elderly

As chronic conditions increase the vulnerability to heat stress (2,6), a reduction of co-morbidity will reduce heat-related mortality. UGS have a proven effect on cardiovascular disease, obesity, type II diabetes and mental health (3,7), conditions known to contribute to heat vulnerability (8).

**Social interaction and participation**

- IPT 5: By increasing social interaction and participation UGS increase the heat responsiveness of elderly

Social interaction between neighbours is considered to be a mechanism behind the relationship between greenspace and health (9). Social capital is mentioned as being protective for heat-related mortality (8). Social isolation may lead to a delay in treatment for heat stress (2). As UGS enhance social interaction, social capital (3,7), this way it can reduce heat-related mortality.

**Bibliography**

1. Santamouris M, Osmond P. Increasing green infrastructure in cities: Impact on ambient temperature, air quality and heat-related mortality and morbidity. Buildings. 2020;10(12):1–34.

2. World Bank. Analysis of Heat Waves and Urban Heat Island Effects in Central European Cities and Implications for Urban Planning. Analysis of Heat Waves and Urban Heat Island Effects in Central European Cities and Implications for Urban Planning. 2020.

3. Dadvand P, Gidlow C, Kruize H, Grazuleviciene R, Lawrence R, Maas J, et al. Positive health effects of the natural outdoor environment in typical populations in different regions in europe (phenotype) project. Epidemiology [Internet]. 2012;23(5 SUPPL. 1):S576. Available from: http://ovidsp.ovid.com/ovidweb.cgi?T=JS&PAGE=reference&D=emed12&NEWS=N&AN=71289917

4. Fritsch N, Kreis G, Matthies F. Improving Public Health Responses To Extreme Weather Events - Meeting Report. 2008;22–3.

5. Revi A, Satterthwaite DE, Aragón-Durand F, Corfee-Morlot USA J, Kiunsi RB, da Silva J, et al. Urban Areas. In: : Climate Change 2014: Impacts, Adaptation, and Vulnerability Part A: Global and Sectoral Aspects Contribution of Working Group II to the Fifth Assessment Report of the Intergovernmental Panel on Climate Change. Cambridge; 2014. p. 535–612.

6. WHO. Urban green spaces: A brief for action [Internet]. Regional Office For Europe. 2017. Available from: http://www.euro.who.int/__data/assets/pdf_file/0010/342289/Urban-Green-Spaces_EN_WHO_web.pdf?ua=1

7. WHO. Urban green space interventions and health: A review of impacts and effectiveness. WHO Reg Off Eur [Internet]. 2016;80. Available from: http://www.euro.who.int/pubrequest%0Ahttp://www.euro.who.int/pubrequest%0Ahttp://www.euro.who.int/__data/assets/pdf_file/0005/321971/Urban-green-spaces-and-health-review-evidence.pdf?ua=1

8. Kovats RS, Hajat S. Heat stress and public health: A critical review. In: Annual Review of Public Health [Internet]. Annu Rev Public Health; 2008 [cited 2021 Dec 20]. p. 41–55. Available from: https://pubmed.ncbi.nlm.nih.gov/18031221/

9. WHO Regional Office for Europe. Urban green spaces and health. Copenhagen; 2016.
