# Supplementary material for: How do urban green spaces influence heat-related mortality in elderly? A realist synthesis
Source: BMC Public Health. 2024 Feb 13;24:457. doi: 10.1186/s12889-024-17973-5 (PMC10865713; doi:10.1186/s12889-024-17973-5)
Supplement: Supplementary file 2 — Supplementary Material 2: Annex B: List of articles with evidence for CMOC’s [file 12889_2024_17973_MOESM2_ESM.docx]

## ANNEX B: List of articles with evidence for CMOC’s

Table S1: articles used as evidence for the CMOC's
