# Supplementary material for: How do urban green spaces influence heat-related mortality in elderly? A realist synthesis
Source: BMC Public Health. 2024 Feb 13;24:457. doi: 10.1186/s12889-024-17973-5 (PMC10865713; doi:10.1186/s12889-024-17973-5)
Supplement: Supplementary file 3 — Supplementary Material 3: Annex C: Selected evidence in literature for CMOC’s [file 12889_2024_17973_MOESM3_ESM.docx]

## ANNEX C: Selected evidence in literature for CMOC’s

1. **CMOC 1**

**Proximity of UGS is important for elderly**

Distance to green spaces was a relevant factor for the elderly indicating a preference for short access times. Lack of trees along the street leading to the green space was clearly disliked. The factor of ‘street benches’ was not signiﬁcant.

On the other hand, access played a more important role for the elderly during heat days compared to previous ﬁndings on adults’ green-space preferences in Vienna (Arnberger and Eder, 2015): long access through heatexposed streets may discourage them from a visit of even shady and attractive green spaces.

(Arnberger et al., 2017)

In contrast, other studies have emphasized the importance of the proximity of parks. For example, Ravenscroft and Markwell (2010) concluded that urban parks and open spaces remain predominantly local facilities. The results of their study showed that the majority of people traveled for less than 15 min to visit their local park. Coles and Bussey (2000), who investigated the criteria used by the public to value urban woodlands in the UK, concluded that woodlands should ideally be located within 5 min walking of the home. Also, Gobster (1995) based on a study on greenway trail perception and use, advised planners to develop local trails so that they can be reached from a distance of 5 miles or less. Speciﬁcally, for older adults a service radius of 1 mile may be needed to make the trails reasonably accessible.

Moreover, as people age they in general face a declining health and decreasing activity spaces making them more dependent on their direct living environment for their activities (e.g., Rantanen et al., 2012; Schwanen & Paez, 2010).

(Kemperman & Timmermans, 2014)

The subgroup analyses show that the relation between green space and loneliness and the relation between green space and shortage of social support was strongest in the strongly urban areas. Furthermore, as we hypothesised, both relations appeared to be strongest for children and elderly who, as a consequence of limited mobility rely more on their neighbourhood to support their needs (Kweon et al., 1998).

(Maas et al., 2009)

Quantitative evidence suggest that the most obvious environmental factors, such as quality and proximity of UGS, are comparatively weak influences (Schipperijn et al., 2010

(Picascia & Mitchell, 2022)

**Accessibility of UGS is important for elderly**

Participants reported that access routes between home and UGSs were not designed

and maintained effectively. In some neighbourhoods, the design of park features, pedestrian infrastructure, and the streetscape did not meet the needs of older adults with mobility difﬁculties. Lack of safe walking infrastructure (e.g., unobstructed footpaths, non-slip surfaces, street lighting) and age-friendly facilities (e.g., benches, toilets) in parks were barriers to the use of UGSs.

*“I like to go to the park at the end ofmy street, but it is difficult because it does not serve my needs. I suffer from joint pain and need to sit down frequently. There are no resting spots, benches, or toilets, so I find it difficult when I am there.” (Male, Age 72, NewDelhi)*

*“The park in my neighbouhood is very popular with children and younger people because it has many facilities for them. There is a badminton court and volleyball net. But, for older people like me, there is nothing. Not even proper benches with a backrest and arms or non-slip surfaces to walk. When it rains, it is so risky. One ofmy friends slipped and hurt herself.” (Female, Age 65, Chennai)*

*“The footpaths are no longer places where people can walk. They have become parking bays for cars. Without the park, I don’t know where I would walk safely and go for exercise. It is impossible to walk on the streets these days. They are ﬁlled with motorcycles, cars, autorickshaws leaving no space for us.” (Female, Age 66, Chennai)*

For UGSs to beneﬁt the health of older adults, the quality and design of the features may need to be adapted. In our study, features such as benches, shaded walking paths with non-slip surfaces and outdoor lighting were especially important. For older adults to move around, walkability, connectivity, and safety of streets around the UGS, and access to public transport are key factors. The quality of UGS is dependent on its integration with neighbourhood planning, design of streetscapes, and pedestrian infrastructure [5,6].

(Adlakha et al., 2021)

It is known that the availability of a bench or a seat for resting purposes is important for the elderly (Jorgensen and Anthopoulou, 2007; Rodiek and Fried, 2005), although in several studies the presence of benches did not predict the elderly’s preferences for green spaces or walking attractiveness of streets (Arnberger and Eder, 2011; Borst et al., 2008). The non-signiﬁcant inﬂuence of the street bench in this study indicates that the majority of elderly residents – even the home-coping segment – perceived themselves as ﬁt enough and not needing to rely on benches along green spaces access streets

Moreover, as people age they in general face a declining health and decreasing activity spaces making them more dependent on their direct living environment for their activities (e.g., Rantanen et al., 2012; Schwanen & Paez, 2010).

(Kemperman & Timmermans, 2014)

The home-coping segment reported poorest health status, the lowest number of people living in the household and was least satisﬁed with its neighbourhood (Table 3). The outdoor-coping segment reported highest health status and lives in small apartments frequently in districts with lower socio-economic status. This segment was more satisﬁed with neighbouring green spaces, which it visited more often than other segments. The second-home coping segment lives in largest apartments, with two-thirds living in higher socioeconomic districts. Of all the segments, they were least satisﬁed with neighbouring green spaces.

(Arnberger et al., 2017)

The focus group research, which provided a rich description of features (and barriers to) making communities age-friendly, formed the basis for an age-friendly guide and checklist (WHO, 2007 ). For instance, in terms of outdoor spaces, focus group participants identiﬁed a clean, safe environment and green space as assets, and uneven sidewalks and unsafe pedestrian crossings as barriers.

(Menec et al., 2011)

First, as the possibility for older people to enjoy parks as a non-discriminant environment when they become frailer because health issues can restrict older people’s park use in cases of insuﬃcient facilities and equipment, e.g., benches and toilets (Alidoust and Bosman, 2015).

The inclusive design of urban green spaces such as parks relates to a number of particular infrastructure elements and facilities, including pleasant vegetation, trees for shade, age-convenient benches, toilets, shelters from extreme weather situations (e.g., heat) and paved trails for older people (Arnberger et al., 2017; Aspinall et al., 2010; Knight et al., 2018). Ottoni et al. (2016) highlighted that benches are particularly needed to motivate older people to visit parks. Benches should be designed so that older people can sit comfortably with adapting height and shape to adjust for potential physical limitations. Gibson (2018) highlighted that benches should allow older people to rise from the bench on their own – with arm and back rests – and be installed in a way that allows people to cluster in order to enable social interaction. In addition, safety is an important neighbourhood characteristic that was considered to be a prerequisite for older people’s use of urban green spaces (Alidoust and Bosman, 2015; O’Brien, 2014). Safety may be ensured through an inclusive urban design that considers the safe interaction of diverse user groups, e.g., pedestrians and cyclists (Knight et al., 2018) and protection from nuisances as well as potential adaptations to the physical built environment to ensure that disabled people can cross roads safely or even access a park with ramps instead of steps (O’Brien, 2014).

(Enssle & Kabisch, 2020)

**Order and safety are important for elderly**

Neighbourhood disorder (e.g., vandalism, grafﬁti, litter) constrained older adults’ use of UGS. Older women, in particular, reported feeling unsafe while walking through UGSs and vulnerable to anti-social behaviour. Some older adults avoided UGSs because of crime, signs of physical disorder, and threats to personal safety.

*“The park is only used by groups ofmen to drink alcohol. It is not safe for women so I avoid it. They are always loitering around, hanging around at the entrance to the park. I don’t even feel like going there. It is so unpleasant.” (Female, Age 62, New Delhi)*

*“I work as a cleaner all seven days ofthe week. Sometimes, I walk through the park which is on the way, but there is no proper lighting on the paths. When I leave for work in the mornings, it is still dark and most ofthe time, the street lights are not working, or someone has broken them. This is the only chance I have to walk in a green space and escape from the traffic and noise, but I don’t feel safe in the park.” (Female, Age 61, Chennai)*

(Adlakha et al., 2021)

Hung and Crompton (2006) identiﬁed leisure constraints associated with the use of an urban park reported by elderly people in Hong Kong. Among the major constraints was crowding. The elderly are particularly concerned about the presence of dogs and incivilities such as aggressive visitor behaviour in public spaces (Arnberger and Eder, 2011; Robin et al., 2007

(Arnberger et al., 2017)

Women are found to be underrepresented in the use of green spaces compared to men (e.g., Hutchison, 1994), while in general they spend more time in their neighborhood (Kavanagh, Bentley, Turell, Broom, & Subramanian, 2006). The fear of violence and the perception that a green space is unsafe is a barrier to women to use the green space

Wright Wendel, Zarger, and Mihelcic (2012) investigated perceptions and behavior of green space users in a rapidly urbanizing city (Santa Cruz, Bolivia), with a speciﬁc interest in gender and socio-economic inequalities. They also found that barriers impacting green space access were found to be most signiﬁcant for women, lower-income, and less centrally located residents, and that this ﬁnding was related to whether the green spaces were perceived as safe

In her study, she concluded that inner-city parks need to be well-maintained and safe in order to realize their full potential in supporting social interactions and developing social ties. The issues of safety and maintenance were also addressed by a number of other studies (e.g., Humpel et al., 2002; Michael, Green, & Farquhar, 2006). Oh (2003) found that a low safety level in the neighborhood is associated with low social cohesion because residents are less likely to participate in outdoor activities. Speciﬁcally, it was concluded that elderly, with their weak physical capability and decreasing self-efﬁcacy, withdraw from social contacts with neighbors, with increasing anxiety about unsafe and uneasy neighborhood conditions. Moreover, older people that live in a well-maintained neighborhood tend to remain independent for a longer period (Sugiyama & Ward Thompson, 2006; Wentzel, Rose, & Rockwood, 2001).

The maintenance of green spaces also affects the perceived level of green and is an important aspect associated with the safety in the living environment. This is in line with a study by Bedimo-Rung et al. (2005) who concluded that badly maintained green spaces may fall into disrepair and lower the visual qualities of the setting, implicating that less social behavior are accepted. Perceived safety levels may diminish and stimulate crime and vandalism rates. Also Matsuoka and Kaplan (2008) concluded that through properly designed urban spaces social interactions can be promoted.

(Kemperman & Timmermans, 2014)

Safety can also be thought of as being associated with the social environment. For example, fear of crime and perceptions of lack of safety relate to the larger socioeconomic environment of a community (e.g., Clark et al., 2009 ). A sense of safety can also arise from the availability of well-maintained sidewalks and stairs that have railings – both of which are aspects of the physical environment. Thus, safety might more usefully be thought of as an outcome, or result, of agefriendly features rather than an aspect of the community environment per se

(Menec et al., 2011)

However, people who fear being robbed and who are afraid to leave their dwelling, limit their outdoor physical activities, such as walking (Ross, 2000).

(Young et al., 2004)

Social well-being may not be beneﬁcially aﬀected by green and open space that is perceived as unsafe or where people engage in antisocial behavior, although proper management and maintenance can address these problems. There is also some evidence that the provision of new green spaces in disadvantaged neighborhoods (e.g., greening of vacant lots) can reduce crime [1]

(Kruizse et al., 2019)

**Feeling of belonging of feeling at ease with other visitors is important for elderly**

In the social domain, focus group participants commented, among many other things, on the need for bringing generations together and fostering a culture of respect for older adults.

(Menec et al., 2011)

The perceptions subgroups (such as adults and young people) have of one another can lead to self-exclusion of some (such as adults) from parks. This self-exclusion becomes an important barrier to greenspace access not captured in understandings of physical quality of greenspace. For Archie (below) accessibility was impeded by the presence of younger people.

*‘They could make it a bit more accessible, I don’t mean getting there, but I mean it’s inhabited by the youngsters now and they’re not very friendly youngsters, you know, it can be quite intimidating at times, you know. I think perhaps if they put more effort into maybe policing that park then you’d probably get people having a walk round it. I would rather travel anyway, you know, for a walk I would rather travel somewhere else, you know.’ Archie, more deprived area*

These responses appear to have origins in the level of integration and confidence individuals felt in their communities.

Previous research has identified the presence of others as a key facilitator of greenspace use, particular for women however, a decline in trust in communities can make the presence of others a barrier to use and is not always interpreted positively [41

(Seaman et al., 2010)

Perceived differences with other users, particularly in terms of SES, affected the reported willingness of respondents to visit a certain green space, with some of the interviewees identifying the presence of neds (a derogatory Scottish term referring to someone of a low social standing) as cause for avoiding specific spaces. Similarly, Gibson (2018) found the presence of people of “similar social class” as a factor considered by older Australian adults when deciding whether to visit UGS.

(Picascia & Mitchell, 2022)

Research on natural open spaces shows that cultural disposition and behavioural codes are key factors that discourage minority ethnic communities from using those spaces (Morris, 2003). In addition, many empirical studies (e.g. McDonald and McAvoy, 1997; Gobster, 1998) have found strong evidence for the presence and the relevance of discrimination.

(Peters et al., 2010)

In addition, older people have been found to closely relate the physical accessibility of their neighbourhood with social components, such as knowing their neighbours and feelings of familiarity (Menec et al., 2011; Van Dijk et al., 2015). These ﬁndings suggest that social integration could serve as a prerequisite for older people to visit nearby public spaces, such as urban parks

(Enssle & Kabisch, 2020)

Most people understand the boundaries between public and private space and have quite precise notions about which forms of behavior or activities can be performed where, how and when. This feeling for in- and out-of-place behavior is thus not just a question of state discipline and government regulation, but equally part of a shared cultural background, social control and sense of place. It is now generally recognized that the design and organization specific places or buildings (see Gieryn 2002, Lees 2001, Dovey 1999) involves persuading or affirming people to act in accordance with certain expectations of what is considered ‘good and proper’. It involves the construction of a script with built-in norms and values, but also legal rules and legislation. According to Cresswell, “order is inscribed through and in space and place.” (Cresswell, 1996, 55)

(Haan, 2005)

**Perception and awareness are important**

Perceived safety in the neighborhood was also related to someone’s satisfaction with his or her social network. The higher the perceived level of safety, the more satisﬁed with their network and conversely, a low level of perceived safety was in line with a lower satisfaction with the social network.

(Kemperman et al., 2019)

Safety can also be thought of as being associated with the social environment. For example, fear of crime and perceptions of lack of safety relate to the larger socioeconomic environment of a community (e.g., Clark et al., 2009 ). A sense of safety can also arise from the availability of well-maintained sidewalks and stairs that have railings – both of which are aspects of the physical environment. Thus, safety might more usefully be thought of as an outcome, or result, of agefriendly features rather than an aspect of the community environment per se

(Menec et al., 2011)

Finally, it is important to avoid over-regulating design and space, as security and well-being are more likely to grow out of active use (Mean and Tims, 2005)

(Peters et al., 2010)

Several studies indicate the importance of combining the actions outlined above to increase the use of urban green space. Thus, actions targeting quantity and quality of green space (“hardware”) may be combined with actions that promote awareness of availability, location, accessibility of local green space (“software”).

(Kruizse et al., 2019)

**Deprived neighbourhoods, los SES has negative effects**

‘Low-SES neighborhoods also provide fewer activity-friendly public open spaces and are less walkable than high-SES neighborhoods [13,16,17]’

‘

Studies of parks have reported less frequent use, poorer perceived accessibility and safety, and poor perceived distance to public parks in more deprived areas [18,19].’

(Schmidt et al., 2019)

Several studies have demonstrated that the beneﬁcial associations between greenspace and health are strongest for those with low individual-level SES and those residing in more deprived neighbourhoods (Dadvand et al., 2012a, 2012c, 2014b; de Vries et al., 2003; Maas et al., 2009b; McEachan et al., 2016; van den Berg et al., 2016). One explanation for these variations is that those with low SES generally have a worse health status and live in more polluted areas, which makes them more likely to beneﬁt from a health promotion intervention (Bolte et al., 2010; de Vries et al., 2003; Su et al., 2011). A second explanation is that those with low SES are less mobile and consequently, spend more time near their home, which makes them more dependent on their immediate greenspace (Maas, 2008; Schwanen et al., 2002). The opposite appears true for people with high SES (Bell et al., 2010; Greenspace Scotland, 2008).

(Markevych et al., 2017)

‘Studies of parks have reported less frequent use, poorer perceived accessibility and safety, and poor perceived distance to public parks in more deprived areas [18,19].’

(Schmidt et al., 2019)

1. **CMOC 2**

**UGS are seen as a place of comfort**

Participants reported lower levels of exposure to noise, air pollution, and heat in UGSs. For many older adults, UGSs offered respite and escape from toxic exhaust fumes from motorized vehicles and polluted air in the city.

(Adlakha et al., 2021)

During heat periods, 55% of the respondents stay at home (the so-called ‘home-coping segment’), 31% go outside (‘outdoor-coping segment’) and 14% visit their second home (‘second-home coping segment’; Table 2). Most of the home-coping segment feel that it was hotter outside, believe it to be too exhausting to go out mainly due to age or generally prefer to stay at home. The outdoorcoping segment mostly visits green spaces, followed by indoor places such as churches and blue spaces.

(Arnberger et al., 2017)

Indeed, as noted by Ward Thompson and Aspinall [25] “natural open space offers opportunities for peace, relaxation, and social activities and, for many, physical activity is a secondary benefit, rather than a primary purpose in visits”.

(Adlakha et al., 2021)

**UGS as a place for physical activity**

A study in Florida found that the accessibility of green spaces, but not the mere amount of green space, was associated with decreases in all-cause mortality and mortality from cardiovascular diseases [14]. A Danish study found that usability, as indicated by the presence of walking and cycling routes, was positively related to physical activity in the nearest green space, but not to physical activity levels in general [24]. These latter findings suggest that relationships between green space availability and public health involve more than an increase in general physical activity levels. Indeed, as noted by Ward Thompson and Aspinall [25] “natural open space offers opportunities for peace, relaxation, and social activities and, for many, physical activity is a secondary benefit, rather than a primary purpose in visits”.

(Adlakha et al., 2021)

Hence, even though social interaction might not occur with walking in older adults within NOS, it seems to be a vital part for the elderly’s quality of life, as their social gatherings in the NOS give them a reason to get out of their apartments, which on the other hand makes them walk, as they have to leave the apartment to meet friends and neighbors in NOS. If so, walking may not occur extensively within the NOS, but on the way to the NOS, and NOS as such, are more important for social interaction than for walking.

**UGS are seen as meeting places**

As most of the interviewed older adults lived alone, these social outdoor spaces in their immediate surrounding seem to be especially important for them. As one interviewed man put it: “ . . . well a lot of people are alone right . . . but then they meet down there (by the benches and raised beds) and talk . . . ” This quote highlights the importance of outdoor social spaces in order for older adults to maintain social engagement and counteract the loneliness that is often associated with aging.

As many older adults face loneliness due to loss of their partner and friends, social relations may be especially important for this age group. One study by Yung et al. [69], investigating older adults living in urban renewal districts in Hong Kong, stressed that older adults consider social spaces and activities as their most important needs, rather than walkable and safe open spaces. This is further conﬁrmed in another study by Yung et al. [70], who stressed the need to focus more on social spaces when planning and designing public parks, and to include the population in decision making

(Schmidt et al., 2019)

A study in Florida found that the accessibility of green spaces, but not the mere amount of green space, was associated with decreases in all-cause mortality and mortality from cardiovascular diseases [14]. A Danish study found that usability, as indicated by the presence of walking and cycling routes, was positively related to physical activity in the nearest green space, but not to physical activity levels in general [24]. These latter findings suggest that relationships between green space availability and public health involve more than an increase in general physical activity levels. Indeed, as noted by Ward Thompson and Aspinall [25] “natural open space offers opportunities for peace, relaxation, and social activities and, for many, physical activity is a secondary benefit, rather than a primary purpose in visits”.

(Zhang et al., 2015)

The focus group research, which provided a rich description of features (and barriers to) making communities age-friendly, formed the basis for an age-friendly guide and checklist (WHO, 2007 ). For instance, in terms of outdoor spaces, focus group participants identiﬁ ed a clean, safe environment and green space as assets, and uneven sidewalks and unsafe pedestrian crossings as barriers. In the social domain, focus group participants commented, among many other things, on the need for bringing generations together and fostering a culture of respect for older adults.

(Menec et al., 2011)

The quantitative survey showed that social interaction is valued by both non-Western migrants and native Dutch people, whether that interaction is with the people with whom they visit the park or with other, known or unknown people. This conclusion was conﬁrmed by the qualitative survey. Both native Dutch people and non-Western migrants see urban parks as places where they can meet other people: they want to spend time with their friends and family and to meet other people. However, in most cases meeting ‘other people’ meant meeting people they knew. Nevertheless, most visitors do talk to strangers in urban parks, though most conversations are rather short and relate to either speciﬁc issues – such as children and dogs – or to everyday issues, like the weather. Furthermore, although people do not visit parks to meet strangers, they do like to engage in small talk with new acquaintances.

(Peters et al., 2010)

**UGS are seen as a place to go with dogs and grandchildren**

Surveys show that those who own dogs visit green spaces much more frequently (Burnett et al., 2021),

(Picascia & Mitchell, 2022)

*“I like to go to the park at the end ofmy street, but it is difficult because it does not serve my needs. I suffer from joint pain and need to sit down frequently. There are no resting spots, benches, or toilets, so I find it difficult when I am there.” (Male, Age 72, NewDelhi)*

*“The park in my neighbouhood is very popular with children and younger people because it has many facilities for them.*

(Arnberger et al., 2017)

Not so much now no I don’t [use the local park] I probably will because I’m going to have a grandchild soon.

(Seaman et al., 2010)

“I have a big extended family and bring my grandchildren to this park. The big advantage is that there is a play area for kids and a few benches for older people like me to sit. I have managed to meet other grandparents and we have a nice community now. All our grandchildren play with each other.”

(Adlakha et al., 2021)

**Park use will depend on the design and infrastructure**

A growing body of research in non-pandemic periods suggests that the physical features of green space inﬂuence people’s use behaviors. For instance, green space size (35), proximity (36), maintenance (12), facilities (37), and aesthetic features such as vegetation (38), water (12), and sound (39) are physical factors that are closely related to green space visitation.

(Li et al., 2021)

Park use and the recognized ecosystem services provided by a park clearly depend on the park structure and infrastructure. The Friedenspark, with its high tree coverage, the lawn areas and more satisfying flora and fauna, is used for natural experiences significantly more frequently, while Lene-Voigt-Park, with its open areas, many sports, playing and seating facilities, is used for socializing as well as having BBQs and picnics significantly more often. In the summer heat, the different park characteristics and usages may complement each other to serve different recreational demands of different user groups at different times of day. Kabisch et al. (2020) showed that different age groups also preferred dedicated areas in parks with specific characteristics. They found that young children frequented playgrounds and lawns, while older people preferred to sit on benches.

Activity differences can also be related to the design of the parks, as design facilitates certain activities and limits others. The quantitative results show that walking and meeting other people are least popular in the Haarlem urban park. In the Arnhem urban park, cycling is less popular and having a picnic or barbecue is very popular; the slopes make it less attractive for cycling, while the panoramic views make it very attractive for having a picnic or a barbecue

Apart from the differences in park use that can be linked to the cultural preferences of varied ethnic groups, use differences in Nijmegen can also be related to park design. Goffertpark has an open character: it has a large and open meadow, which is very attractive for all kinds of activities. Non-Western migrants enjoyed a picnic more often than native Dutch people, and in all cases had food and drinks with them. The open character also stimulates active leisure activities: the space is used intensively for active forms of recreation such as playing football, running and skating. This park is also used by families with small children, who play with balls and other objects. This results in an active and busy atmosphere. Thiemepark, on the other hand, is much more quiet. Native Dutch people also tend to eat in this park; however, this was a rather speciﬁc group, namely students. Besides, hardly any houses in this district have gardens; therefore people use the park as their back garden, which is why native Dutch people eat in this park. People also visit the park to relax, to talk and to meet people. When Thiemepark was designed, one of the clear objectives was to create a meeting place. The grassy area is therefore not ﬂat but sloping; playing football in this newly designed park is not encouraged. The differences in park design lead to different usage and consequently to different ambiences.

The interviews revealed some of the possible reasons why Thiemepark offered more familiarity. The design process of Thiemepark was much more inclusive of local residents. They had asked for a green place in the park where they could sit, relax and meet each other, and the design reﬂects many of these wishes. Sitting the meadow on a slope was meant to discourage the playing of football and thus minimize possible conﬂicts in use. Rest and gathering are supposed to be the main functions of this park. In addition, the wish to have water in the park was expressed by Moroccan people. Furthermore, the multicultural character of the neighbourhood was explicitly acknowledged, for example by including Arabic elements, such as a sculpture by an Arab artist

(Peters et al., 2010)

Aspinall et al. (2010) used a conjoint choice experiment to investigate the relative importance of various attributes of neighborhood or local parks for older adults. Results indicated that lack of nuisance, presence of facilities, trees and plants were the most important park attributes.

(Kemperman & Timmermans, 2014)

Prior studies identified important attributes of UGS, which are regarded as important predictors of UGS use (Bedimo-Rung, Mowen, & Cohen, 2005; Van Herzele & Wiedemann, 2003). However, other studies exhibited inconsistent results, such as those in Schipperijn, Stigsdotter, Randrup, and Troelsen (2010) who failed to identify a reliable predictor of UGS use. A. Y. Lo and Jim (2010) found that UGS quality is not correlated with visit frequency. Therefore, the important attributes of UGS are yet to be clearly and comprehensively identified (Maruani & Amit-Cohen, 2007). Schipperijn et al. (2010) suggested that these inconsistent findings are due to contextual differences of the studies.

(Wan & Shen, 2015)

**Perception is important**

Objectively demonstrable conditions (such as provision of infrastructure and greenspace) are experienced through subjective and inter-subjective ‘rationalities’ around the appropriateness of using greenspace as a leisure choice or in daily life.

(Seaman et al., 2010)

These different ambiences are also created by the function and the image of urban parks. The large-scale Goffertpark functions as a city park and is famous in Nijmegen. Most of our respondents had been visiting it for quite some years. They characterized the park with expressions like ‘seeing other people and being seen’. Thiemepark is a small neighbourhood park, suitable for everyday use. Most of the visitors live nearby and know each other from other places in their neighbourhood. More people greet each other here than in Goffertpark. The park can be described as the back garden of the visitors and as an ordinary everyday place.

(Peters et al., 2010)

Some behaviors seemed to be more motivated by outdoor temperatures versus indoor temperatures. For example, the behavior of leaving the house, based on outdoor temperature, steadily increased over the pre-determined temperature intervals. This suggests that the perception of the weather being hotter – e.g., based on media reports – could encourage a person to leave the house, more so than the actual temperature indoors.

(White-Newsome et al., 2011)

Again, vegetation seems to have a positive impact on people’s perception of heat that goes beyond its simulated cooling effect. We suggest that due to previous experiences and people’s general knowledge that plants and trees provide shade and coolness, visual stimuli can provoke those very sensations. The same effect occurs with water. Where urban vegetation is scarce, water takes on an important cooling function, even if it is not “used” in a strict sense. Blue infrastructure (in our cases the lake and the fountains) seems to be able to compensate for the lack of green infrastructure, to some extent (Figure 7).

Our results support the idea that urban vegetation not only reduces objective heat loads but also reduces feelings of crowding and increases (thermal) well‐being. This is in line with other studies that have found positive psychological effects of vegetation for thermal comfort (Klemm et al., 2015; Nikolopoulou & Steemers, 2003), urban stress (Kabisch et al., 2021; Knöll et al., 2018), and health (Kondo et al., 2018).

(Mittermüller et al., 2021)

First of all it can be concluded that perceived greenness of the living environment is of major importance for the social contacts between the residents living in this neighborhood. The perceived greenness has a moderating role between the availability of the green space types grass and trees, and social contacts among aging neighbors in their living environment

(Zhang et al., 2015)

In general, the results revealed that UGS components aﬀected the perceived health beneﬁts. However, people’s use behaviors in UGSs were more likely inﬂuenced by individual characteristics rather than UGS components. Furthermore, place attachment partially mediated the relationship between UGS components and perceived health beneﬁts but fully mediated the association between UGS components and use behaviors during the COVID-19 pandemic

(Li et al., 2021)

by using urban parks people connect to certain places and to the other people who use those places. This can lead to social cohesion because it promotes feelings of comfort. Dines and Cattell (2006, p. ix) conclude that ‘‘commitment to the local area and its people often inﬂuenced the use and experience of public open spaces, and so too were positive perceptions of spaces and the valued opportunities they afforded for casual social encounters’’. Also De Haan (2005) shows that the appropriation of public places can lead to more attachment to these places and more possibilities to make contact, and thus to more social cohesion.

(Peters et al., 2010)

That Moroccans pay fewer visits to urban parks than Turks might be explained by the fact that the Turkish community is very close-knit and has a strong park culture, implying that going to natural areas, especially with families, is more a part of Turkish culture than of Moroccan culture.

(Peters et al., 2010)

**Needs are important for (perceived) usefulness**

Some behaviors seemed to be more motivated by outdoor temperatures versus indoor temperatures. For example, the behavior of leaving the house, based on outdoor temperature, steadily increased over the pre-determined temperature intervals. This suggests that the perception of the weather being hotter – e.g., based on media reports – could encourage a person to leave the house, more so than the actual temperature indoors.

(White-Newsome et al., 2011)

Green space was more important to older adults living in high-rise apartments and multi-occupancy residential dwellings.

(Adlakha et al., 2021)

*‘We do not have space inside our homes, so the playground is the only space to get a breath of fresh air. I wish the municipality would improve its quality. Everything is broken and damaged. Even a few benches for older people to sit will help.” (Male, Age 64, New Delhi)*

*“I used to work as a housekeeper and was very active until I was 60, but then I had a stroke and one side ofmy body was affected. The doctor recommended physiotherapy, but I could not afford it long-term. The gym equipment in the nearby park is the only free option I have to exercise, but some ofit is broken and unusable.”*

(Adlakha et al., 2021)

Wanka et al. (2014) analysed stated behaviour of elderly residents during heat episodes living in and off heat islands of Vienna and found that the majority stayed in their apartments when it was hot because they perceived their homes to be cooler than the outdoors. Others went outdoors, mostly visiting green spaces. Similarly, Klinenberg (2002) observed that the elderly often stayed at home during the Chicago heat wave in 1995 because of less attractive and safe neighbourhoods and because of lacking social neighbourhood ties. These studies suggest that green spaces can be an important refuge for urban residents during heat periods if they are attractive, safe and cooler

(Arnberger et al., 2017)

The home-coping segment reported poorest health status, the lowest number of people living in the household and was least satisﬁed with its neighbourhood (Table 3). The outdoor-coping segment reported highest health status and lives in small apartments frequently in districts with lower socio-economic status. This segment was more satisﬁed with neighbouring green spaces, which it visited more often than other segments. The second-home coping segment lives in largest apartments, with two-thirds living in higher socioeconomic districts. Of all the segments, they were least satisﬁed with neighbouring green spaces.

(Arnberger et al., 2017)

Similar to previous studies on urban green space and street pref- erences (Arnberger and Eder, 2011, 2015; Borst et al., 2008; Hung and Crompton, 2006; Sugiyama and Ward Thompson, 2008), the elderly in this study prefer easily accessible green spaces which are quiet and attractive. If green spaces provide shade, blue spaces and a cooler environment than the homes during heat periods, the majority of older people will visit those areas. Compared to a preference study in the urban context (Arnberger and Eder, 2011), the elderly speciﬁcally placed importance on heat-related factors, while social factors such as visitor numbers were less important.

(Arnberger et al., 2017)

The preferences of the members of the home-coping segment were inﬂuenced by their health and social status. Temperature differences between green space and indoor environments strongly affected the likelihood of a visit. Despite retreating to their apartments, a heat-adjusted design of a nearby cool green space would encourage up to 85% of them to visit a green space. Nevertheless, 15% would not leave home probably due to their health status and limited mobility. Consequently, the likelihood of visiting green spaces depends on individual factors and on on-site social factors as well because they clearly dislike green spaces with no people. One can speculate whether they are in need for social stimulation or prefer other on-site visitors because of safety reasons

(Arnberger et al., 2017)

Several studies have demonstrated that the beneﬁcial associations between greenspace and health are strongest for those with low individual-level SES and those residing in more deprived neighbourhoods (Dadvand et al., 2012a, 2012c, 2014b; de Vries et al., 2003; Maas et al., 2009b; McEachan et al., 2016; van den Berg et al., 2016). One explanation for these variations is that those with low SES generally have a worse health status and live in more polluted areas, which makes them more likely to beneﬁt from a health promotion intervention (Bolte et al., 2010; de Vries et al., 2003; Su et al., 2011). A second explanation is that those with low SES are less mobile and consequently, spend more time near their home, which makes them more dependent on their immediate greenspace (Maas, 2008; Schwanen et al., 2002). The opposite appears true for people with high SES (Bell et al., 2010; Greenspace Scotland, 2008).

(Markevych et al., 2017)

Conditions of the living environment such as a high-density living have been linked to poor social relations in a variety of communities (e.g., Kuo et al., 1998; Keane, 1991). Kazmierczak (2013) stated that green spaces are particularly important in high density, inner city areas, as urban green space in dense city areas is a limited resource (Kragsig Peschardt & Karlsson Stigsdotter, 2013).

(Kemperman & Timmermans, 2014)

Planetary health crises such as the COVID-19 pandemic have illustrated the interdependencies of human beings and natural ecosystems. During periods when virus containment measures were being implemented, the pandemic clearly showed the benefits of close-by green spaces for people using them to participate in safe outdoor activities with their households and families and to practise sports to maintain their health and well-being (Venter et al., 2020; Xie et al., 2020).

(Kabisch et al., 2021)

Not so much now no I don’t [use the local park] I probably will because I’mgoing to have a grandchild soon.

(Seaman et al., 2010)

Finally, the relation was strongest for people with a low income or a low level of education, indicating that they beneﬁt most of green space in their living environment for their social contacts. Apparently, the amount of green space in the living environment is less important for social contacts of people with a high SES. The studies conducted in Chicago also found a relation between social ties and the presence of green public space for people with a low SES (Coley et al., 1997; Kuo et al., 1998; Kweon et al., 1998). However, because these studies were conducted in an underprivileged area in Chicago, no comparison could be made between the relation for lower and higher SES-groups

(Maas et al., 2009)

All activities are important to both non-Western migrants and native Dutch people. However, it became clear that the activities ‘having a picnic or a barbecue’ and ‘meeting other people’ are much more important to non-Western migrants than to native Dutch people, whereas ‘walking’ and ‘cycling’ are more or less equally important to both groups. In addition, Moroccan people use urban parks more often for meeting other people than do Turkish people.

(Peters et al., 2010)

If we take a closer look at the activities of the ethnic groups in the qualitative study, our observations show that native Dutch visitors cycle and walk more often than do non-Western migrants. Dutch people with dogs come on a daily basis, while non-Western migrants mainly come on Sundays and when the weather is ﬁne; they come to the park with family and friends and spend their time in the shade of the trees. Although both groups use the urban ARTICLE IN PRESS K. Peters et al. / Urban Forestry & Urban Greening 9 (2010) 93–100 parks for social gathering, eating and relaxing, non-Western migrants tend to visit urban parks in groups, whereas native Dutch people tend to visit in small groups, as couples or alone. This difference can be explained by the fact that most nonWestern migrants put more value on their families. Especially the leisure behaviour of Muslims can be characterized as collectivistic in nature and with a strong focus on family ties.

(Peters et al., 2010)

Apart from the differences in park use that can be linked to the cultural preferences of varied ethnic groups, use differences in Nijmegen can also be related to park design. Goffertpark has an open character: it has a large and open meadow, which is very attractive for all kinds of activities. Non-Western migrants enjoyed a picnic more often than native Dutch people, and in all cases had food and drinks with them. The open character also stimulates active leisure activities: the space is used intensively for active forms of recreation such as playing football, running and skating. This park is also used by families with small children, who play with balls and other objects. This results in an active and busy atmosphere. Thiemepark, on the other hand, is much more quiet. Native Dutch people also tend to eat in this park; however, this was a rather speciﬁc group, namely students. Besides, hardly any houses in this district have gardens; therefore people use the park as their back garden, which is why native Dutch people eat in this park. People also visit the park to relax, to talk and to meet people. When Thiemepark was designed, one of the clear objectives was to create a meeting place. The grassy area is therefore not ﬂat but sloping; playing football in this newly designed park is not encouraged. The differences in park design lead to different usage and consequently to different ambiences.

(Peters et al., 2010)

‘having a picnic or a barbecue’ and ‘meeting other people’ are much more important to non-Western migrants than to native Dutch people. This supports the conclusion of Elmendorf et al. (2005) that social motives are less important for whites than for African-Americans. This can be related to the collectivistic nature of leisure behaviour combined with the importance of family for non-Western migrants (Stodolska and Livengood, 2006).

(Peters et al., 2010)

1. **CMOC 3**

**People can feel comfortable and relaxed in UGS**

A few studies have investigated the relationship between green spaces within neighborhoods and social interaction for older adults, and found that social interaction is inﬂuenced by the availability of trees, grass and greenness of the green space, along with safety and maintenance [40–42].

Another study found seating to be important for older adults’ walking behavior within the neighborhood [59]. Another reason could be that there is usually more activity in places that include seating, as they attract diﬀerent groups of people to sit and talk, rest, read a book, and have a picnic. This might be especially attractive for older adults, as they can be socially isolated, thus, walking in a neighborhood with open space with lots of people and activity may be more interesting for older adults, as they may feel safer, which previously has been found to be important [32], less alone and more entertained with the possibility of talking to other community members [60]. As one resident mentioned; “But I greet them all, because now I know them . . . they wave at me . . . and we exchange some words . . . ”.

(Brewster et al., 2019)

Natural settings in common space are attractive because they can for example provide shadow, privacy and sound buffering from surrounding environments and they could have restorative effects (Coley et al., 1997; Hartig et al., 2003; Kaplan and Kaplan, 1989). Three closely related studies, performed by the same research group in an underprivileged area of Chicago, provide indication of a positive relation between the presence of green public facilities and social ties (Coley et al., 1997; Kuo et al., 1998; Kweon et al., 1998).

(Maas et al., 2009)

If the physical characteristics of the neighbourhood are not conducive to establishing contact, then obtaining support may be more difﬁcult, particularly for older people who live alone (Thompson & Krause, 1998). Such physical barriers, as well as psychological barriers such as the fear of crime, can result in fewer opportunities for contact and support.

(Young et al., 2004)

**UGS can be perceived as a meeting place**

Another study found seating to be important for older adults’ walking behavior within the neighborhood [59]. Another reason could be that there is usually more activity in places that include seating, as they attract diﬀerent groups of people to sit and talk, rest, read a book, and have a picnic. This might be especially attractive for older adults, as they can be socially isolated, thus, walking in a neighborhood with open space with lots of people and activity may be more interesting for older adults, as they may feel safer, which previously has been found to be important [32], less alone and more entertained with the possibility of talking to other community members [60]. As one resident mentioned; “But I greet them all, because now I know them . . . they wave at me . . . and we exchange some words . . . ”.

especially the social relationships or casual encounters around seating places with diﬀerent neighbors were important for the residents. As one interviewed woman said: ”We just sit and chat, just for a couple ofhours or three and then we leave again . . . I have brought coﬀee with me and we just sit and enjoy . . . I really don’t want to sit there all by myself”.

(Schmidt et al., 2019)

As most of the interviewed older adults lived alone, these social outdoor spaces in their immediate surrounding seem to be especially important for them. As one interviewed man put it: “ . . . well a lot of people are alone right . . . but then they meet down there (by the benches and raised beds) and talk . . . ” This quote highlights the importance of outdoor social spaces in order for older adults to maintain social engagement and counteract the loneliness that is often associated with aging.

(Brewster et al., 2019)

Participants described new friendly relationships or partnerships developing as a result of impromptu and unplanned interactions in parks. While personal goals or desires were achieved, community building and increased social capital also emerged.

(Adlakha et al., 2021)

“I have been living alone for the past few years. This park has given me an outlet and a chance to meet neighbours during my evening walks. I have made friends here and they check on me if they don’t see me outside for my walk. It gives me comfort knowing that I have a community nearby.” (Male, Age 70, New Delhi

(Adlakha et al., 2021)

In our study, participants reported increased communication and non-familial intergenerational interaction between younger and older adults in UGSs. Successful non-familial intergenerational interactions were achieved through shared experiences and meaningful outdoor activities in UGSs.

(Adlakha et al., 2021)

Bedimo-Rung et al. (2005) concluded that parks or other green spaces may provide a meeting place where people can develop social ties.

(Kemperman & Timmermans, 2014)

green spaces in the living environment can promote social contacts as they provide places where people can meet, greet and talk with other people (e.g., Kabisch & Haase, 2013; Kuo, Sullivan, Coley, & Brunson, 1998; Martin, Warren, & Kinzig, 2004; Peters, Elands, & Buijs, 2010).

(Kemperman & Timmermans, 2014)

Furthermore, research has investigated the socio-economic sta- tus of residents in relation to social contacts and green spaces (e.g., Bedimo-Rung et al., 2005; Coley, Kuo, & Sullivan, 1997; Kweon et al., 1998). These studies suggest that parks and other green spaces in poor urban areas promote increased opportunities for social interaction. This review of the inﬂuence of individual and household char- acteristics suggests that especially age, health status, gender, and socio-economic status account for differences in the use of green spaces, affecting opportunities for social conta

(Kemperman & Timmermans, 2014)

The social contacts with friends and family are related to social contacts in the living environment, but do not seem to be linked to green space availability, these contacts probably take place at home and/or other environments. A study by Dines and Cattell (2006) investigated the signiﬁcance and value of all kind of public spaces for enabling social contact in a neighborhood in East London. They concluded that as well as green spaces, hard spaces such as streets and markets need to be more widely recognized as important public spaces in promoting social contact.

(Kemperman & Timmermans, 2014)

Concerning the relation between green space and social contact, our results show that people with more green space in their living environment feel less lonely and experience less shortage of social support, but they did not have more contact with neighbours or friends in the neighbourhood and they did not receive more social support. This suggests that the relation between green space and social contacts has more to do with the fact that green spaces can strengthen sense of community via place attachment and place identity of its residents, than with actual contacts with neighbours.

(Maas et al., 2009)

Goffman (1963) argues that, as a rule, people do not interact in public spaces unless there is an obvious reason to do so; he calls this ‘civil inattention’ (Goffman, 1963, p. 84). Most of the time we simply follow the social codes of conduct in order to avoid colliding with other people.

Nevertheless, interactions do take place and civil inattention can be broken. An external stimulus can provide a linkage between strangers that leads to social interaction; this is called triangulation (Loﬂand, 1998). In a public space, the choice and arrangement of the various elements in relation to each other can set the triangulation process in motion.

The presence of an event or amenity can draw strangers together.

Interactions can also be created by people who enjoy making contact with others in public spaces trying to pass the time by having a chat, sharing an unexpected experience, getting some information on a topic of interest or basking in the momentary glow of ‘fellow feeling’ (Loﬂand, 1998, p. 39)

Interactions through triangulations were usually triggered by balls, children and dogs. Children played together for a while, parents chatted with each other and balls were returned when needed. These interactions were deﬁned by the visitors as ‘small talk’ and ‘having a chat about a common interest’, and were valued positively.

(Peters et al., 2010)

These different ambiences are also created by the function and the image of urban parks. The large-scale Goffertpark functions as a city park and is famous in Nijmegen. Most of our respondents had been visiting it for quite some years. They characterized the park with expressions like ‘seeing other people and being seen’. Thiemepark is a small neighbourhood park, suitable for everyday use. Most of the visitors live nearby and know each other from other places in their neighbourhood. More people greet each other here than in Goffertpark. The park can be described as the back garden of the visitors and as an ordinary everyday place.

(Peters et al., 2010)

in both parks only a few interactions occurred; visitors were very much on their own. More intensive interactions, such as talking and sitting together, happened only incidentally in both parks. In general, going to the park to meet strangers was not one of the main motivations. Most of the visitors use the park as a kind of back garden. Many of them live near the park; they will often leave the park and come back a short while later with something to drink. As one respondent said: ‘‘People like to be on their own.’’ Nevertheless, people greeted each other more often in Thiemepark people than in Goffertpark

(Peters et al., 2010)

The quantitative survey showed that social interaction is valued by both non-Western migrants and native Dutch people, whether that interaction is with the people with whom they visit the park or with other, known or unknown people. This conclusion was conﬁrmed by the qualitative survey. Both native Dutch people and non-Western migrants see urban parks as places where they can meet other people: they want to spend time with their friends and family and to meet other people. However, in most cases meeting ‘other people’ meant meeting people they knew. Nevertheless, most visitors do talk to strangers in urban parks, though most conversations are rather short and relate to either speciﬁc issues – such as children and dogs – or to everyday issues, like the weather. Furthermore, although people do not visit parks to meet strangers, they do like to engage in small talk with new acquaintances.

(Peters et al., 2010)

We can conclude that most social interactions are cursory, for example, people have a short chat or just say hello. Many of them start with an external stimulus and follow the rule of triangulation. Visitors do not have many intensive social interactions with people they do not know. There are more weak and one-off interactions than strong and more structural interactions. Most of the visitors feel comfortable within their own social group and do not feel the need to interact with others. This is in line with earlier research (cf. Loﬂand, 1998) that concluded that interactions with strangers are less common than those with known people. We also have to take into account that what Goffman (1971) described as ‘norms of civil inattention’ are fundamental to the interaction order. Respectful interactions enable citizens to have rewarding social interactions and to develop social networks that are sustained by trust. These in turn support a wider social sphere that is characterized by peaceful coexistence, prosperity and inclusion

(Peters et al., 2010)

Peters et al. [24] analyzed activities (e.g., walking, cycling, having a barbecue, or a meeting) that may stimulate social interactions and social cohesion. While their observations varied by park location and sociodemographic variables (e.g., Dutch and non-Western migrants), urban parks were viewed as a place for social gatherings and other leisure activities

(Jennings & Bamkole, 2019)

The presence of green spaces in a neighbourhood motivates older people to be more physically active (Mytton et al., 2012; Sugiyama and Thompson, 2007; Takano et al., 2002), with positive impacts on cardiovascular health (Astell-Burt et al., 2016) and mental health (Lee and Lee, 2019; Thompson Coon et al., 2011), particularly as part of community-based programmes (Barton et al., 2012) and through the integral provision of opportunities for social interaction (Aspinall et al., 2010).

(Enssle & Kabisch, 2020)

Levasseur et al., 2017). Knight et al. (2018) showed that older people mostly visit parks with others, particularly with their partners. Accordingly, our results showed that most of the older people who frequently used parks were married. This ﬁnding suggests that social integration is a precondition for older people to visit parks at all.

(Enssle & Kabisch, 2020)

Alidoust and Bosman (2015) showed that a close neighbourhood environment with green spaces can be considered a particular arena for fostering social ties between older people and enable an experience of neighbourliness (Alidoust and Bosman, 2015; Knight et al., 2018). In this sense, urban green spaces can be regarded as “spaces of encounter” (Piekut and Valentine, 2017; Valentine, 2008), which are important to enable social contacts, to meet other people and to engage with strangers (Neal et al., 2015; Peters, 2010; Peters, 2010).

(Enssle & Kabisch, 2020)

**The design, facilities and activities organised in UGS can promote social gathering and shared experiences**

Matsuoka and Kaplan (2008) reviewed articles to explore the insights into how humans interact with outdoor environments. One of their conclusions was that through properly designed urban spaces improved social interactions can be promoted.

(Kemperman & Timmermans, 2014)

“I have a big extended family and bring my grandchildren to this park. The big advantage is that there is a play area for kids and a few benches for older people like me to sit. I have managed to meet other grandparents and we have a nice community now. All our grandchildren play with each other.”

(Adlakha et al., 2021)

Community gardens created avenues for informal gathering older adults took ownership of the space, collectively grew plants, and developed friendships. Some participants reported working on a community project or common goal with others in the neighbourhood.

(Adlakha et al., 2021)

“There was a neglected corner of the park that was becoming a garbage dump. One of my friends decided to clean it up and plant shrubs and ﬂowers. I also joined her, and then many others joined us. We now take turns in watering the plants. It has become a collective effort.” (Female, Age 66, New Delhi) “My neighbour and I started a community garden near the park. We grow vegetables, herbs, and ﬂowers. We also started a small gardening group where we share tips and exchange plants with others. This has now grown to over 75 participants. It has given me something to look forward to and keeps me busy.” (Female, Age 60, Chennai) Social and cultural events were often held in parks and gardens, providing opportuni- ties for older adults to interact and develop social bonds. “There is a shrine in the centre ofthe park managed by the neighbourhood association. Every morning, there is a puja (an act ofworship) attended by many park visitors. Many people bring ﬂowers and garlands they have made themselves and also make rangoli (decorative ﬂoor patterns). On auspicious days and festivals like the New Year or Diwali, we have a big gathering here and everyone celebrates together.” (Male, Age 62, Chennai) “On Children’s Day, a group ofus . . . retired grandparents organise outdoor cultural events for children in the park. Last year, we had face painting and many outdoor games like hopscotch and tug ofwar.” (Female, Age 65, New Delhi)

(Adlakha et al., 2021)

In our study, women reported participating in group-based physical activity programmes and community projects with new friends as a result of increased social interactions in their local UGS. Studies have found that women who frequently exercised in a group developed a sense of familiarity with each other and therefore felt safer [34]

(Adlakha et al., 2021)

In our study, participants reported increased communication and non-familial intergenerational interaction between younger and older adults in UGSs. Successful non-familial intergenerational interactions were achieved through shared experiences and meaningful outdoor activities in UGSs.

(Adlakha et al., 2021)

Sullivan, Kuo, and Depooter (2004) concluded in their study that that by spending more time in outdoor green spaces with trees and grass residents get to know their neighbors better, leading to more social interaction in the neighborhood. Todorova, Asakawa, and Aikoh (2004) explored preferences for various street planting models, with different compositions of ﬂowers and with or without trees. Flowers were the most preferred elements and also seem to have a positive inﬂuence on psychological well-being. While, Peters et al. (2010) speciﬁcally focused on whether urban parks can facilitate social cohesion in urban neighborhoods. They concluded that parks facilitate all kind of outdoor activities such as walking, cycling, playing, having a picnic or a BBQ, and also meeting other people

(Kemperman & Timmermans, 2014)

Heat resilience of social activities varies significantly compared with necessary and optional activities. In public spaces with strong supportive land uses and planned events, social activities are as heat-resilient as necessary activities. Examples are Federation Central Plaza, and Darling Quarter. However, in the absence of supportive facilities and planned events, social activities are extremely heat-sensitive and begin to disappear immediately after NTTout.

(Sharifi & Boland, 2017)

frequently walking in the neighborhood could lead to more spontaneous social interactions [41],

(Kemperman et al., 2019)

trees and grass in common spaces, as opposed to barren common spaces, may attract residents to outdoor spaces, thereby leading to more frequent contacts among neighbours (Coley et al., 1997).

(Maas et al., 2009)

A study by Ewert and Heywood conducted in the US (1991) showed that undertaking activities in natural environments appeared to have stimulating effects on social contacts and social cohesion. The results of a study by Leyden (2003) show that people in Ireland in walkable neighbourhoods, which are among other things characterised by the availability of local parks, are ‘more likely to know their neighbours, to participate politically, to trust others, and to be involved socially’

(Maas et al., 2009)

Thus, urban parks are sites where different ethnic groups mingle. They are sites where informal and cursory interactions occur and with which people feel connected. Insight into the function, image and design of a park is crucial for understanding the extent to which these places can facilitate social cohesion. Urban parks that function as everyday places are places in which people feel at home. Visitors can easily connect to the place and to other visitors, because many visitors will already be familiar with each other from the neighbourhood. Urban parks that function as a ‘world of strangers’ attract a variety of people. People feel welcome because these urban parks are open and accessible. Although the functions of parks differ, both types provide a vital locality where everyday experiences are shared and negotiated with a variety of people. Organizing activities can stimulate interactions. Learning from the functions of and practices in urban spaces may help to make non-urban green areas more inclusive.

(Peters et al., 2010)

The following factors may relate urban green spaces to social interactions: an open park design to encourage active recreational activities [24], the availability of sidewalks [27], improved access to parks through quality transportation options [28], shaded areas that support relaxing environments [24], functional playgrounds [29], and the extent of organized activities [30]. Hence, characteristics of the built environment and amenities near urban green spaces maybe associated with social cohesion [31]. These studies also imply that the level of engagement within the green space (e.g., environmental stewardship and other volunteering) can vary based upon qualities of the green space (e.g., access and amenities), the intended use (e.g., for leisure and recreation), and an area’s overall social context.

(Jennings & Bamkole, 2019)

Providing safe spaces for people to meet and socialize together with the provision of social community programmes that encourage elderly people to go out (Knight et al., 2018) and visit friends and neighbours have been shown to be eﬀective strategies in promoting and increasing physical activity patterns (Chaudhury et al., 2016). Because older people may regard social contacts in the neighbourhood as a prerequisite for park visitation, community programmes may also involve other spaces of encounter, such as neighbourhood cafés, libraries or community gardens.

**If elderly engage in social contact will depend on if they feel they belong, feel connected with or accepted by other visitors**

Perceived differences with other users, particularly in terms of SES, affected the reported willingness of respondents to visit a certain green space, with some of the interviewees identifying the presence of neds (a derogatory Scottish term referring to someone of a low social standing) as cause for avoiding specific spaces. Similarly, Gibson (2018) found the presence of people of “similar social class” as a factor considered by older Australian adults when deciding whether to visit UGS.

(Picascia & Mitchell, 2022)

We can conclude that most social interactions are cursory, for example, people have a short chat or just say hello. Many of them start with an external stimulus and follow the rule of triangulation. Visitors do not have many intensive social interactions with people they do not know. There are more weak and one-off interactions than strong and more structural interactions. Most of the visitors feel comfortable within their own social group and do not feel the need to interact with others. This is in line with earlier research (cf. Loﬂand, 1998) that concluded that interactions with strangers are less common than those with known people. We also have to take into account that what Goffman (1971) described as ‘norms of civil inattention’ are fundamental to the interaction order. Respectful interactions enable citizens to have rewarding social interactions and to develop social networks that are sustained by trust. These in turn support a wider social sphere that is characterized by peaceful coexistence, prosperity and inclusion

(Peters et al., 2010)

**The need for social contact is important**

However, the World Health Organization (2011) stated that in

countries with very low birth rates, future generations will have fewer, if any siblings. The global trend toward having fewer chil- dren induces less potential family care and support for the elderly. Gardner (2011) showed that although the family has been iden- tified as the most important source of informal support there is increasing awareness of the important role of non-family support. In particular, friends and neighbors increasingly contribute to the well-being of older adults. Moreover, Volker, Flap, and Lindeberg (2007) indicated that social contacts in the neighborhood are more likely to emerge if residents have few alternatives. Furthermore, they concluded that a typical living environment that affects an individual’s social contacts consist of no more than two or three streets in the direct vicinity of the home. Therefore, this research focused at this scale, the direct living environment of the aging population.

(Kemperman & Timmermans, 2014)

**Link with social participation and social cohesion**

Our study demonstrated the transformation of community liabilities into assets. In both cities, community gardens were established through informal interaction with neighbours and social networks. Studies of the social dynamics of community gardens have illustrated the relationships between urban greening and community building [39,40]

(Adlakha et al., 2021)

Participants described new friendly relationships or partnerships developing as a result of impromptu and unplanned interactions in parks. While personal goals or desires were achieved, community building and increased social capital also emerged.

(Adlakha et al., 2021)

“I have been living alone for the past few years. This park has given me an outlet and a chance to meet neighbours during my evening walks. I have made friends here and they check on me if they don’t see me outside for my walk. It gives me comfort knowing that I have a community nearby.” (Male, Age 70, New Delhi

(Adlakha et al., 2021)

“I have a big extended family and bring my grandchildren to this park. The big advantage is that there is a play area for kids and a few benches for older people like me to sit. I have managed to meet other grandparents and we have a nice community now. All our grandchildren play with each other.”

(Adlakha et al., 2021)

We can conclude that most social interactions are cursory, for example, people have a short chat or just say hello. Many of them start with an external stimulus and follow the rule of triangulation. Visitors do not have many intensive social interactions with people they do not know. There are more weak and one-off interactions than strong and more structural interactions. Most of the visitors feel comfortable within their own social group and do not feel the need to interact with others. This is in line with earlier research (cf. Loﬂand, 1998) that concluded that interactions with strangers are less common than those with known people. We also have to take into account that what Goffman (1971) described as ‘norms of civil inattention’ are fundamental to the interaction order. Respectful interactions enable citizens to have rewarding social interactions and to develop social networks that are sustained by trust. These in turn support a wider social sphere that is characterized by peaceful coexistence, prosperity and inclusion

The results from Thiemepark showed that a combination of knowing other visitors and having cursory interactions leads to feelings of comfort and makes people feel at ease. This is in line with Dines and Cattell (2006), who conclude in their research in South-east London that these cursory interactions facilitate feeling at ease in certain public places. Although results from Thiemepark indicate that informal and cursory interactions can stimulate social cohesion (cf. Kleinhans et al., 2007), the results from the other urban parks do not indicate a direct relation between interaction and social cohesion.

(Peters et al., 2010)

Milligan et al. points out the importance of communal gardening to combat social isolation and a supportive community environment helping less able members of the group

(Brewster et al., 2019)

participation in park organizations can led to stronger perceptions of social cohesion

(Jennings & Bamkole, 2019)

strong social cohesion can encourage positive interactions that facilitate participation in clubs and organizations

(Jennings & Bamkole, 2019)

For example, studies have highlighted that older participants in group-based nature conservation and gardening activities appreciate the opportunities gained for structure and routine; meaningful social interaction and the development of stronger communities; a sense of achievement, pride and ownership; and the forging of new social identities (e.g., [85]). In addition, van den Berg et al. found that allotment gardeners of 62 years and older scored better on all measures of health and well-being than neighbors in the same age category without an allotment garden, living next to the home addresses of allotment gardeners [86].

(Kruizse et al., 2019)

Coll-Planas et al.’s [75] study into prevention of social isolation and loneliness in the older people through weekly group activities found that after two years, 39.5% still participated in the activities, almost half maintained contact with each other, and overall they reported improvement in mood and social wellbeing. Similarly, Harada et al.’s study [76], led by Kobe University, found that older people’s social network improved signiﬁcantly following a year-long, 18 theme-based programs aimed at promoting social interactions.

(Vu et al., 2019)

'They found that women were more involved in strong face-toface local networks, often with other women, while men were more involved in non-local networks. The same study (13) found that women were generally acknowledged as those who ‘create local community’ and this was possibly steered by gender expectations of women as primarily responsible for the home and living environment. We believe that women’s greater involvement in bridging social networks may be a result ofexisting gender relations with higher expectations that women should be involved, for example, in children’s activities.'

(Eriksson, 2011)

1. **CMOC 4**

**Certain (perceived) features of a place can make it easier for people to become attached**

The presence of urban green spaces may encourage the developed of place attachment. For example, during a social assessment of urban parks in Jamaica Bay (New York City), researchers found that urban parks foster social interactions, enhanced place attachment, and social resilience [95]

(Jennings & Bamkole, 2019)

Certain physical features of a place can make it easier for people to become attached to that place [39]. For instance, in a case study in a retirement community, physical features such as close walking distance to the central activity building and better access to outdoor garden space were found to be essential physical features of place attachment, because they may support social interactions [40]. In terms of green spaces, their availability may be of key importance for people to foster a sense of attachment. However, limited research has tied place attachment to the availability of green space, and measured the impact of green space attachment on health and wellbeing.

(Zhang et al., 2015)

Research has shown that the majority of people’s favorite places consist of natural places [41]. These findings may be interpreted as a manifestation of biophilia, or an innate predisposition to affiliate with natural places and other life-like processes [42,43]. Given people’s strong tendency to connect with nature, it seems appropriate to distinguish “green space attachment” as a special form of place attachment that is highly significant to people.

(Zhang et al., 2015)

Natural features can promote a sense of community by increasing feelings of emotional attachment to a neighbourhood and people’s identity with a place, which in turn could decrease feelings of loneliness and increases social support (Pretty et al., 1994; Prezza et al., 2001).

(Maas et al., 2009)

**Participation in planning and design can help**

Participation in the planning process of, for example, a nearby park, may foster feelings of belonging and identiﬁcation and, in turn, enhance park visitation

(Enssle & Kabisch, 2020)

**People have to feel safe to become attached and the other way around**

The level of perceived safety in the neighborhood had a signiﬁcant inﬂuence on the attachment with the neighborhood

(Kemperman et al., 2019)

**Knowing other people in the park fosters a sense of belonging**

Visitors to Thiemepark feel very much at home in this park. The fact that they know quite a number of people when they visit the park gives them a sense of familiarity. They feel comfortable and relaxed. They also like living in the neighbourhood and the idea that most of the visitors come from their neighbourhood.

(Peters et al., 2010)

Most people understand the boundaries between public and private space and have quite precise notions about which forms of behavior or activities can be performed where, how and when. This feeling for in- and out-of-place behavior is thus not just a question of state discipline and government regulation, but equally part of a shared cultural background, social control and sense of place. It is now generally recognized that the design and organization specific places or buildings (see Gieryn 2002, Lees 2001, Dovey 1999) involves persuading or affirming people to act in accordance with certain expectations of what is considered ‘good and proper’. It involves the construction of a script with built-in norms and values, but also legal rules and legislation. According to Cresswell, “order is inscribed through and in space and place.” (Cresswell, 1996, 55)

(Haan, 2005)

The feeling of a place, and the capacity or willingness to read and understand spatial signs, symbols is not self evident, however. According to Holloway and Hubbard (2001), places are always struggled over and contested “the production, occupation and control of place is caught up in an ongoing struggle between different groups and individuals” It is apparent that some social groups have more power than others to determine what is desir- able and where and that the struggle for power over place is taking place both at the political/legal level and at the level of everyday, diffuse forms of power exercise. According to Holloway and Hubbard (2001), in the complex struggle for sense of place “the often taken-for-granted nature of place is revised and reworked.” The order that is imposed ‘from above’ is not only challenged, but also lived and transformed at ‘street level’ where “individuals and groups create their own geographies, using places in ways very different than bureaucrats and administrators intend.”

(Haan, 2005)

This result is in line with a pre-pandemic study suggesting that place attachment has a strong inﬂuence on park utilization and behavioral tendencies (95). People’s emotional connection with others and attention to place can translate into an aﬃnity for the shared environment in which they live

(Li et al., 2021)

The quantitative study explored the extent to which residents feel attached to their local park. It appears that in general people feel reasonably attached (6.5 on a 10-point scale; 1=not attached at all, 10=extremely attached). Native Dutch people, however, are more attached than Turkish and Moroccan people (7.1, 6.1 and 4.6, respectively; eta2=0.14 (Po0.001)). There is no difference between ﬁrst- and second-generation migrants. With respect to other socio-demographics, there are not that many differences, although it is interesting to observe that the older people are, the more attached they are.

(Peters et al., 2010)

Beyond mechanisms derived from the role relationships on which social ties are based, connections to other people are sources of a sense of belonging (Barrera 2000; Berkman 1995; Cobb 1976; Cutrona and Russell 1990; Thoits 1985; Uchino 2004). Belonging implies acceptance and inclusion by members of one’s primary or secondary groups. Acceptance and inclusion are not automatically granted by group members; they must signal their acceptance of us as a part of the group. With acceptance comes a belief that one “belongs to a network of communication and **mutual obligation**”

(Thoits, 2011)

**The length of stay in a neighbourhood and the frequency of visits to UGS are important**

The home and the search for secure, known places can be considered as spaces for accomplishing ontological security (see for instance Dupuis and Thorns 1988 and Easthope, 2004). David Seamon (1979) argues that appropriation is associated with emotional attachment to place and a sense of threat sentiment. It is mostly applied to spaces with a high intensity of use and attachment and provides a person a place of ownness and order in a wider world that is public, often chaotic. Neighborhood appropriation can be seen as an attempt to extend this sentiment of ‘ownness’ to the public sphere of residential space (see Brunson et al. , 2001 on the creation of defensible space).

(Haan, 2005)

Importantly, we found that frequency of visits to neighborhood green space is significantly positive related to green space attachment. A case study in the Netherlands also found that the more often people visit an urban park, the more connected and attached they are to it [58]. Our data also show that older residents are more likely to attach to green space than younger people. This may be because older people are less mobile than young people, and may have more time to visit the green spaces in their neighborhood.

(Zhang et al., 2015)

Women who had lived longer at their current address had a better sense of belonging to their neighbourhood, as did women living in non-urban areas and who were better able to manage on their income. These ﬁndings are consistent with previous studies that showed residential stability to be associated with community attachment (Bolan, 1997). The positive association between length of time in a neighbourhood and feelings of belonging is consistent with the principle that a time lag between exposure and outcome is plausible in social epidemiology (Blakely & Woodward, 2000)

(Young et al., 2004)

1. **CMOC 5**

**Social capital, with its norms of trust and reciprocity lead to neighbourly support**

Understanding the effect of the social environment on the well being of older adults is important for the promotion of active aging in the community. To our knowledge, we are the first to show that in addition to social capital of individuals and the quality of neighborhood services, neighborhood social capital , and social cohesion are significantly and independently associated with well being of older adults. Social cohesion and social capital among neighbors may lead to higher levels of well being in older adults because higher levels of neighborhood cohesion result in higher degrees of social organization, including the provision of instrumental support to neighbors (e.g., support in times of sickness and help with transportation, groceries, picking up mail, and throwing away garbage). These seemingly small favors among neighbors may prevent worries about the future — neighbors take care of each other and watch over each other — that translate into better well being outcomes.

(Cramm et al., 2013)

increased social contacts are a pathway **to receive help with personal interests** [58], high neighborhood cohesion can lead to more social organization and neighborly support (e.g., picking up mail and assistance with transportation

(Jennings & Bamkole, 2019)

In addition to inﬂuencing hospital readmission through the community-level processes described, the norms of trust and reciprocity represented by social capital also foster social network ties for individuals.33 Neighbor-to-neighbor assistance to address social needs may contribute to avoidance of readmissions.

(Brewster et al., 2019)


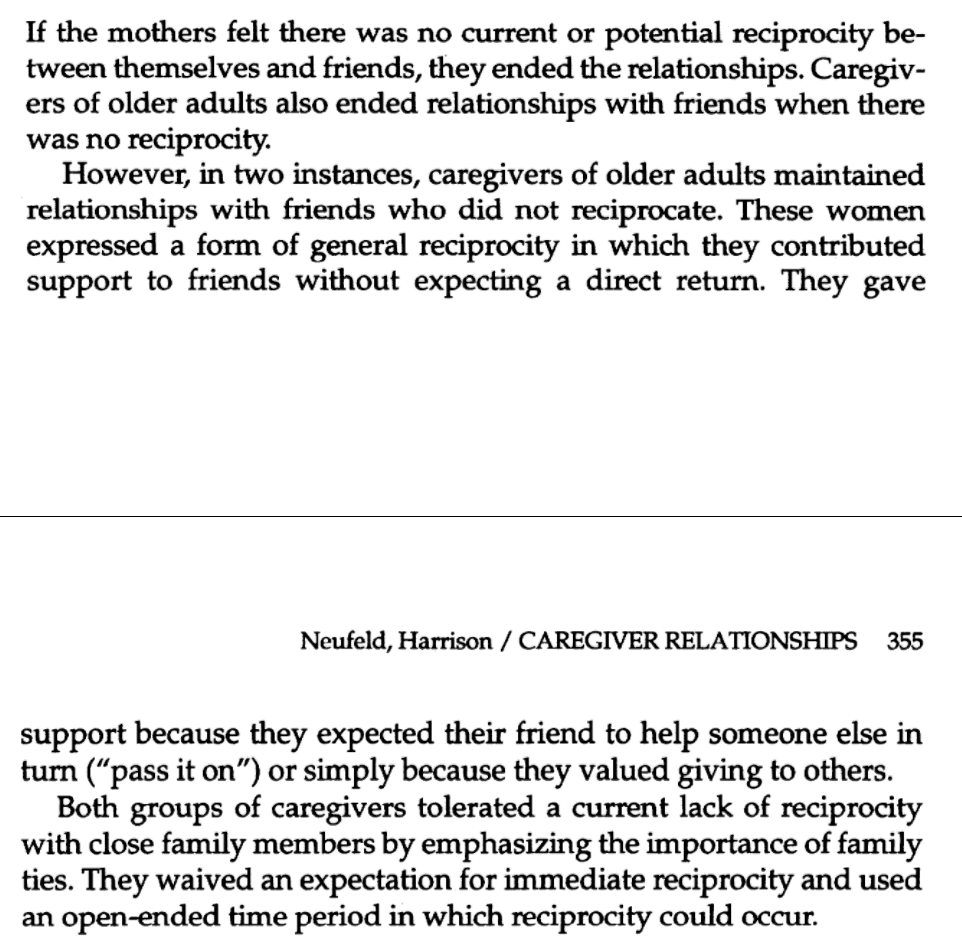


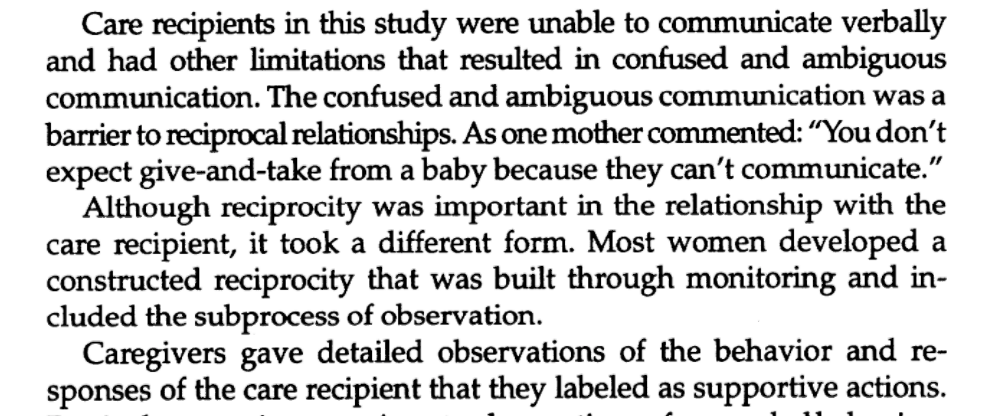


(Neufeld & Harrison, 1995)

**Family is important for social support, but also neighbours are**

In addition to inﬂuencing hospital readmission through the community-level processes described, the norms of trust and reciprocity represented by social capital also foster social network ties for individuals.33 Neighbor-to-neighbor assistance to address social needs may contribute to avoidance of readmissions.

(Brewster et al., 2019)

I have made friends here and they check on me if they don’t see me outside for my walk. It gives me comfort knowing that I have a community nearby.” (Male, Age 70, New Delhi

(Adlakha et al., 2021)

Gardner (2011) showed that although the family has been identiﬁed as the most important source of informal support there is increasing awareness of the important role of non-family support. In particular, friends and neighbors increasingly contribute to the well-being of older adults. Moreover, Volker, Flap, and Lindeberg (2007) **indicated that social contacts in the neighborhood are more likely to emerge if residents have few alternatives**. Furthermore, they concluded that a typical living environment that affects an individual’s social contacts consist of no more than two or three streets in the direct vicinity of the home. Therefore, this research focused at this scale, the direct living environment of the aging population.

(Kemperman & Timmermans, 2014)

The neighbourhood may become a vital element of the support system for older people who become socially isolated through poor health, limited mobility, ﬁnancial constraints or lack of access to transport (Russell et al., 1998).

(Young et al., 2004)

There are indications that the importance of non-kin within the

framework of informal care is on the rise. Barker (2002) showed that other family members, friends and neighbours are increasingly tak- ing on the role of informal caregiver. Ulmanen and Szebehely (2015) confirm that, in Sweden, since 2000 not only the help provided by children but also the help of friends has increased. There are also indications that older people are increasingly receiving more emo- tional and practical support within the framework of friend-based networks (Suanet & Antonucci, 2017). The contribution made by non-kin to the total of informal care is not yet clear (see, e.g., Siira et al., 2019). A great deal is already known about which factors pro- mote or hinder the provision of informal care (see below), but not yet about the associated differences as a function of social relationship (kin and non-kin).

(de Klerk et al., 2021)

**If social support is given, depends on the perceived need by the caregiver**

Additional contacts from neighbours, family, and friends also provide essential linkages to improve favourable health outcomes. However, social contacts and support systems need to be ‘active’ rather than ‘passive’, as described by Wolf et al. [58]. Since older people value their independence and nominated support people are reluctant to impinge on their independence, it is of paramount importance that any preventative measures be implemented in a timely ongoing manner to ensure maximal eﬃciency and eﬀectiveness [57,58]

**Individuals’ independence, and when and how it is asserted, is an important factor in shaping perceptions of elderly and social contacts about heat risk. Social contacts were aware of and acknowledged the independence of the elderly and, perhaps rightly, valued it. In the circumstances of a heat wave, however, failure to constructively challenge this independence could conceivably contribute to the vulnerability of elderly people**

One social contact was worried about their relative’s illness which was exacerbated by the heat, and had in the past acted on it. Another social contact was concerned about heat risks because ofthe direct experience ofthe elderly having suffered from heat stroke some years ago.

the dominant, although not uniform, perception among social contacts of the elderly seems to be one of resilience and common sense compromised only by serious illness, reduced mobility and/ or disability. Evidence for this is well demonstrated by the exemplary quote ‘‘She’s quite sensible regarding the heat!’’ (Norwich primary respondent, female, age 57, about her 80year-old parent). The quote encapsulates the notion that most elderly people, as long as they are sensible, cope well with heat.

These responses suggest that many elderly perceived dealing with heat as ‘‘common sense’’, and that **additional help would be perceived as unnecessary and possibly patronising**. Indeed, this common perception that responding to heat risks is about being sensible was articulated by one respondent thus: ‘‘I don’t think there’s much you can do about it, I mean just common sense things, like, you know, wear loose clothing and I suppose drink plenty of water, um, stay out of the sun.’’ (London primary respondent, female, age 75). These results are congruent with recent ﬁndings from the US, where the majority of elderly respondents felt that heat was not a risk to them personally (Sheridan, 2007).

(Wolf et al., 2010)

**Social support will not necessarily be for everyone in the neighbourhood**

Studies from the UK (13) and Sweden (14) illustrate the complexity of social capital in local communities, and indicate a need to go beyond Putnam’s ‘romantic’ view of community. Westlund (15) suggests that the knowledge society, where internet communication partly replaces civil association activities, has led to societal fragmentation and consequent changes in social capital. Instead of being a pure public good, **social capital has become a ‘club good’** for diverse subgroups within a community or society.

(Eriksson, 2011)

A systematic literature review (42 papers in total) of the association between social capital and health across countries found significant associations between social capital and health in individual and ecological level studies. In contrast, studies investigating the link between collective social capital and health show inconclusive results (30). Similarly, in a systematic literature review of studies investigating the link between social capital and physical health, Kimand colleagues (24) conclude that the strongest associations are between individual social capital and health, particularly between cognitive components of social capital and self-rated health.

(Eriksson, 2011)

'Further, Portes (4) contributes with valuable insights on the potential negative effects of social capital. The **same ties that benefit members ofa network may also lead to exclusion ofoutsiders**. Strong supporting networks may result in an overloadofdemands on some (particularly successful) group members to make resources available. In addition, group participation necessarily demands a certain level ofconformity that might produce restriction in individual freedom.'

(Eriksson, 2011)

**The type of social capital will influence the type and amount of social support obtained.**

'Another important construct is the distinction between bonding, bridging, and linking social capital. **Bonding** social capital is characterised by strong ties within a network that strengthen common identities and functions as a source of help and support *among members*. **Bridging** social capital is characterized by weaker ties that link *people from different networks* together and become important sources of information and resources (12, 28). Szreter and Woolcock (17) introduced **linking** social capital which consists of vertical ties between people in *different formal or institutionalized power hierarchies*.'

(Eriksson, 2011)

'We found (31) that people with higher education were

more likely to have access to all forms of social capital. This was particularly true for bridging social networks; those with higher education were more than four times more likely to have access to this form of social capital compared to people with basic education. Ziersch (44) also found that those with greater resources and higher education had higher access to social capital in Australian households. According to Bourdieu (5), one could assume that the resources resulting from higher education also facilitate access to social capital.'

(Eriksson, 2011)

1. **CMOC 6**

**It’s not only about trust, also norms are important**


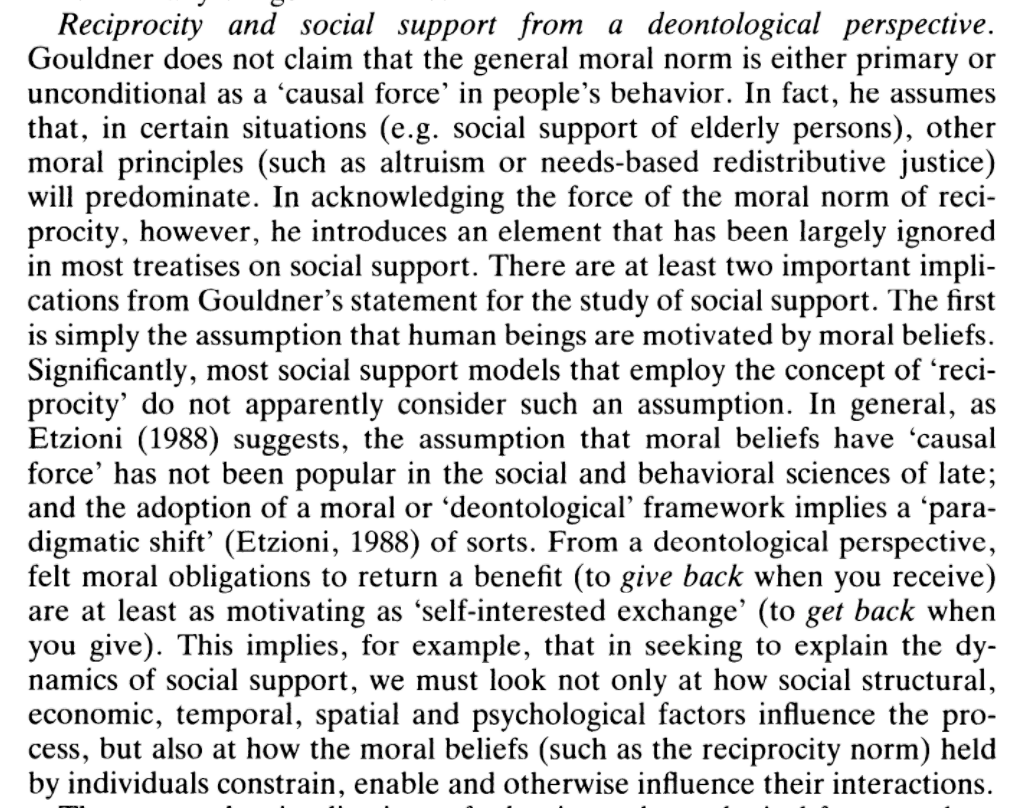


(Uehara, 1995)


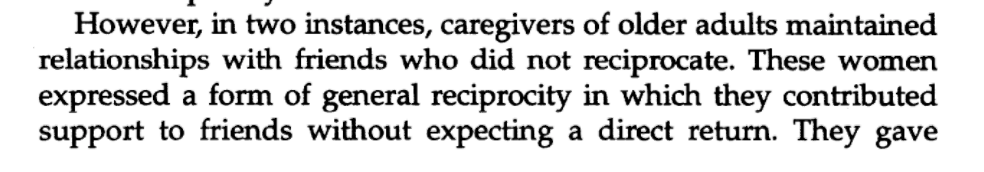


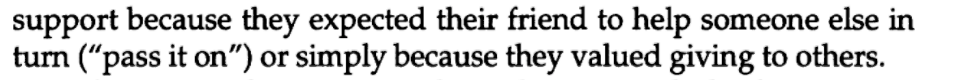


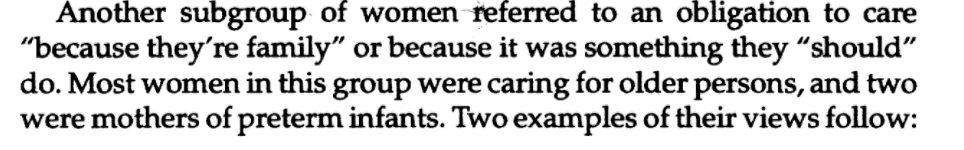

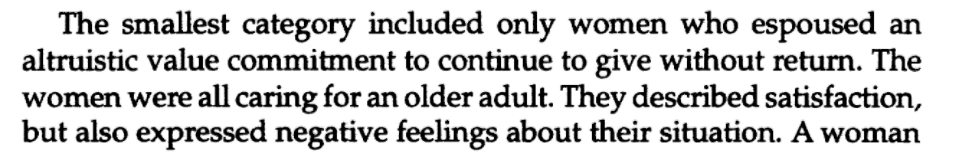


(Neufeld & Harrison, 1995)

﻿The role of social norms is empirically more difficult to assess; they represent by their very nature a less tangible concept than opportunity costs. Analyzing data from SHARE, Klimaviciute et al. (2017) find that, depending on the regions analyzed, informal LTC is mainly driven by a family norm or by moderate altruism. Kotsadam (2011) finds that there is a link between gendered norms and informal care provision by women, and that the strength of this link varies within European countries and is strongest for Germany and Southern European countries. Di Novi et al. (2015) document that female informal care is associated with social norms and cultural traits (see also Costa-Font, 2010 ). 2 Our model explains how gender inequalities in the labor marker and social norms on gender roles affect families’ LTC arrangements, it shows that they reflect an inefficient equilibrium, and studies potentially welfare improving policies

(Barigozzi et al., 2020)

Cross-na- tional comparisons show that in countries with stronger family norms, individuals are more likely to provide informal care (Haberkern and Szydlik 2010; Cooney and Dykstra 2011). A recent study in the Netherlands showed that there has already been a slight shift in recent years towards agreeing that the family carries more responsibility (rather than the government) for providing care in old age (Verbakel 2014). A shift in family-state responsibility is more likely to change the nature of informal care (in hours or types of tasks) and not the decision to care per se, but it is also known that feelings of being needed and obligation increase the likelihood of informal care provision (Oudijk et al. 2011). If the current normative discourse shifts the public view towards more family responsibility and thus strengthens the feelings of being needed, this may con- tribute to more people taking up the caregiver role.

(Broese van Groenou & De Boer, 2016)

When it comes to helping friends or neighbours, young people

help less often compared to people in other age categories. Persons with a higher educational background are more likely to provide help to friends or neighbours. Barriers also play a role: Persons working many hours are less likely to help non-kin compared to people with- out a job. Having experience in the care sector or regularly going to church or mosque make it more likely to help non-kin. There is also a correlation between the norms and giving help: Persons who think that friends or neighbours should help each other more often pro- vide help to these social relations.

(de Klerk et al., 2021)

The study revealed that the long-standing social values, norms, and belief systems of the ethnic group have always prescribed families, extended families, and neighbors to respect and take care of their older members. Nonetheless, poverty in rural households, the diffusion of urban values, and the incipient erosion of longstanding rural values, as well as the tendency of rural youth to abandon agriculture are challenging the capacity of community members to ensure sustained availability of adequate informal care for older adults.

(Alambo & Yimam, 2019)

﻿Existing social capital in this community was characterized by high levels of civic engagement that seemed to be inherited from one generation to the next (14). Strong and dense associations played an important role in getting people involved, and powerful ‘helping-out norms’ obligated people to engage in the community. Strong leaders set the norms and functioned as role models for participation. Effective information channels, e.g. face- to-face meetings, guaranteed that almost everyone was invited to participate.

﻿A second type of extrinsic motivation is introjected regulation. Introjec-

tion describes a type of internal regulation that is still quite controlling be- cause people perform such actions with the feeling of pressure in order to avoid guilt or anxiety or to attain ego-enhancements or pride. Put differently, introjection represents regulation by contingent self-esteem. A classic form of introjection is ego involvement (Nicholls, 1984; Ryan, 1982), in which a person performs an act in order to enhance or maintain self-esteem and the feeling ofworth. Although the regulation is internal to the person, introjected behaviors are not experienced as fully part of the self and thus still have an EPLOC. A more autonomous, or self-determined, form of extrinsic motivation is regulation through identification. Here, the person has identified with the personal importance of a behavior and has thus accepted its regulation as his or her own. A boy who memorizes spelling lists because he sees it as relevant to writing, which he values as a life goal, has identified with the value of this learning activity. Finally, the most autonomous form of extrinsic motivation is integrated regulation. Integration occurs when identified regulations have been fully assimilated to the self. This occurs through self-examination and bringing new regulations into congruence with one’s other values and needs.

(Ryan & Deci, 2000)

1. **CMOC 7**

**People from the same community can learn from each other**

Neighbors live close to each other, and therefore, it is likely that neighbors observe and learn from each other’s behavior [28,29]

(Mohnen et al., 2012)

In contrast, **high social participation may protect against extreme heat as it may increase awareness among friends/family that a person feels ill, and may provide opportunities to seek advice**.2 Promoting social participation among older adults could be an effective strategy to prevent HRHO.

(Laverdière et al., 2016)

**Trusted peers may influence health behaviors in others by functioning as role models**. This influence can be either health-enhancing or health-damaging depending on the existing norms in the network.

Finally, norms and solidarity can make people willing or obliged to participate in various social activities, which can positively influence health through feelings of life meaning, as well as by the achievement of cognitive skills.

(Eriksson, 2011)

high social participation was strongly associated with decreased risk of heat-related EDP and health events. This is consistent with previous studies where increased social contact has been associated with a decreased risk of heat-related mortality12 whereas stopping usual activities during a heat wave has been associated with an increased risk of heat-related morbidity.17 ADL limitation was an independent risk factor of both outcomes. **Loss of autonomy in ADL** has been previously associated with heatrelated mortality.13 Although disability and social participation may be correlated, we observed that they made distinct contributions to HRHO. Needing help in ADL signals an inability to care for oneself, which could lead to difficulties in achieving appropriate actions in order to prevent HRHO. In contrast, **high social participation may protect against extreme heat as it may increase awareness among friends/family that a person feels ill, and may provide opportunities to seek advice**.2 Promoting social participation among older adults could be an effective strategy to prevent HRHO. Age-friendly cities, an approach that supports the creation of active living communities and fosters community engagement,28 could help reach out to isolated older people, who are highly vulnerable to heat owing to a poor health status or potentially unhealthy behaviours contributing to dehydration and impaired judgement. **Low household income** was associated with increased risk of both outcomes, which is consistent with results from a study conducted during the 1999 Chicago heat wave.13 **Low income is associated with financial barriers that restrict a person’s ability to take actions to reduce his or her heat vulnerability (e.g., purchase of air conditioning)**.2

(Laverdière et al., 2016)

We also frequently swap useful information or advice with spouses, neighbors, coworkers, church members, and others as we chat about vexing circumstances encountered in our daily activities (e.g., inconvenient bank hours, mistimed traffic lights, overly expensive goods and services). Facts or recommendations from others enable subsequent behavioral changes that make everyday tasks more efficient, economical, or successful and probably **sustain our sense of mastery or control over life.** Even more importantly, informal discussions with others about impending problems at home, at work, or in other role domains may pinpoint ways to prevent the occurrence of a stressor or de-escalate the appraisal of imminent problems from possible threats to manageable challenges (Cohen and McKay 1984; S. E. Taylor and Aspinwall 1996; Thoits 1985; Uchino 2004).

(Thoits, 2011)

**Social capital will influence risk perception of elderly**

Reviews of social capital indicate that the presence of bridging social capital (links between distinct groups), bonding social capital (relationships between individuals who share social J. Wolf et al. / Global Environmental Change 20 (2010) 44–52 45 identity) or linking social capital (networks of trust across authority gradients) may, albeit not necessarily, lead to an increase in resilience in societies, and that both are associated with survival and recovery from natural disasters (Adger, 2003; Pelling, 2003; Pelling and High, 2005). Cumulated public health evidence raises an expectation that the presence of social capital improves health outcomes and may as a result decrease vulnerability to ill health (e.g. Kawachi et al., 1999; Veenstra, 2000, 2002; Cattell, 2001). But it leaves unclear in which circumstances it may be counterproductive and may increase vulnerability.

Szreter and Woolcock (2004), and indeed literature on risk perception points out that **culturally constructed perceptions in part determine whether a risk is interpreted as threatening, and that perceived risks are highly context speciﬁc** (Slovic and Peters, 2006; Whitmarsh, 2008**). In the context of heat waves it may well be the perceptions ofthose who are at risk from heat stress, and the perceptions of those who provide support and advice to those at risk, that play a key role in inﬂuencing response behaviour, and therefore affect public health outcomes.** Risk perception has both affective-experiential and rational dimensions, and while the two systems interact, the nature oftheir interaction is as yet poorly understood (Slovic et al., 2004). A number of underlying cognitive mechanisms have been well established to govern risk perception and response in other areas (Festinger, 1957; Rachlinski, 2000; Stoll-Kleemann et al., 2001; Keller et al., 2006; Lorenzoni et al., 2007). Cognitive dissonance (realising that the household is at risk while continuing to live in the risk area), availability heuristic (recent events foster easier recall of potential risks), and biased assimilation (knowledge is retained selectively based on related pre-existing beliefs) all operate to prevent a change in practice in individuals.

These results, summarised in Table 2 below, suggest that **in general elderly primary respondents in this research did not perceive themselves at risk from, or vulnerable to, the effects of extreme heat**.

These responses suggest that many elderly perceived dealing with heat as ‘‘common sense’’, and that additional help would be perceived as unnecessary and possibly patronising. Indeed, this common perception that responding to heat risks is about being sensible was articulated by one respondent thus: ‘‘I don’t think there’s much you can do about it, I mean just common sense things, like, you know, wear loose clothing and I suppose drink plenty of water, um, stay out of the sun.’’ (London primary respondent, female, age 75). These results are congruent with recent ﬁndings from the US, where the majority of elderly respondents felt that heat was not a risk to them personally (Sheridan, 2007).

The dominant, although not uniform, perception among social contacts of the elderly seems to be one of resilience and common sense compromised only by serious illness, reduced mobility and/ or disability. Evidence for this is well demonstrated by the exemplary quote ‘‘She’s quite sensible regarding the heat!’’ (Norwich primary respondent, female, age 57, about her 80year-old parent). The quote encapsulates the notion that most elderly people, as long as they are sensible, cope well with heat.

In either case, among both peers and younger social contacts, the primary narrative describes capable, competent and independent elderly people who are well equipped to take care of themselves in the face ofheat risk. Social contacts did not generally challenge the perceptions of their relatives or friends relating to resilience and independence.

As a result, bonding networks act as vehicles of narratives that uphold the lack of prevention and legitimise the reactive approach so prevalent among elderly responses to heat waves. The narratives are founded in perceptions that do not regard heat as a signiﬁcant threat and are constructed by passing information which supports reactive strategies to heat. **Combinedwith a knowledge deﬁcit, and attitudes that encourage independence, these narratives could heighten vulnerability and potentially prevent anticipatory adaptation to the effects of heat waves**

In particular, those networks that lack vertical linkages and linkages to people on the outside of the network, whomay not share similar social identities, have been pointed to as sources of potential conﬂict, albeit this may not be a robust ﬁnding to date (cf. Szreter and Woolcock, 2004).

The bonding networks evidenced in this research probably exist in a context of other types of networks. Individuals interviewed here may engage in other types of social capital which could potentially counteract the narratives transmitted through bonding networks.

The evidence presented here points to a less than straightfor- ward relationship between social capital, vulnerability reduction and increasing resilience. Bonding social capital, depending on context, could play a particular role in shaping vulnerability, potentially even compromising adaptation. **But theoretically it seems that this effect could be counteracted by stronger linking and bridging social capital**. Bridging ties to distinct groups, with different values and perspectives, and linking ties upward through authority gradients could perhaps challenge the views perpetuated by bonding networks.

(Wolf et al., 2010)

Personal perceptions of the health risks of heat are crucial in shaping individual actions to reduce these risks. In previous studies, when people perceive that adaptation to hot weather is unnecessary, they make few to no behavior adjustments to prevent heat-related health risks [3]

Risk perception, fear and self-efficacy are key explanatory factors for protective behaviour of individuals according to Protection Motivation Theory (PMT; Rogers 1975, 1983). In the PMT, risk perception is conceptualised as a combination of the perceived severity and consequences of a threat. Self-efficacy is a central component of coping appraisal and is defined as a person’s perceived ability to carry out a specific action aimed at reducing potential negative consequences (Floyd et al. 2000).

Risk perception, fear and self-efficacy are linked to the social environment: Elliott and Pais (2006, 300) emphasise that ‘people respond to disasters not as isolated individuals but as members of overlapping forms of social affiliation’. Social capital dampens risk perception and fear but increases self-efficacy of households at risk from natural hazards (Wolf et al. 2010; Babcicky and Seebauer 2017).

Socio-demographic variables, such as gender (Babcicky and Seebauer 2017; Kellens et al. 2011), age (Thistlethwaite et al. 2018; Kellens et al. 2011; Zaalberg et al. 2009) and income (Babcicky and Seebauer 2017) have a significant influence on risk perception, fear and self-efficacy.

What is striking is that these collective efficacy components have differing impacts on risk and coping beliefs: risk perception and fear are lowered by social cohesion, but are increased by efficacy belief in citizen groups engaged in preventive participatory action. Social cohesion and group efficacy do not increase self-efficacy, but **belief** in social support does.

Residents with high levels of trust in their community perceive themselves to be at lower risk and therefore may have weaker intentions to prepare for a natural disaster. In a situation of high objective disaster risk, perceiving low risk may lead to a false sense of safety among residents, as discussed in Babcicky and Seebauer (2017). To prevent such an undesired consequence, social cohesion should instead be channelled into hazard-specific collective actions, since the task-specific components of collective efficacy do not seem to bear the risk of counterproductive effects. Belief in social support and in effective citizen group action has the potential to empower residents by making them feel more capable of protecting themselves against natural hazards. If residents believe that they can tackle local risk issues effectively by joining preventive or participatory citizen groups, they tend to be more aware about natural hazards. Here, collective efficacy beliefs may spill over to the individual level, encouraging as well as empowering residents to take risk-reducing actions. Social support and group efficacy together may increase risk perception as well as self-efficacy; this combination is regarded as a pre-condition for protective behaviour (Grothmann and Reusswig 2006).

(Babcicky & Seebauer, 2020)

Social contacts’ knowledge about heat, its health effects and preventive measures was varied and often showed only very broad knowledge of the effects of heat on health. This is relevant because the social contacts were nominated by the elderly respondents as those from whom they would gain advice or assistance and who form the core of their support networks (cf. Wenger, 1992). For example, social contacts did not generally identify dehydration and prolonged exposure to heat overnight as key issues. Despite reporting strategies to keep themselves cool, a number of social contacts identiﬁed few additional ways they could use to help the elderly person to stay cool. Some social contacts implied that their help would be seen as unnecessary and would impinge on the independence of the elderly, a ﬁnding common among spouses. The majority of suggested responses to heat were reactive attempts to reduce its effects when it is already hot. Some of the suggestions could be counterproductive in alleviating heat stress, for example, by encouraging the elderly to drink tea and coffee rather than water or juice. A minority of social contacts bought fans for the elderly person.

(Wolf et al., 2010)

**The provision of correct health information (and other ressources) is important**

Individuals’ independence, and when and how it is asserted, is an important factor in shaping perceptions of elderly and social contacts about heat risk. Social contacts were aware of and acknowledged the independence of the elderly and, perhaps rightly, valued it. In the circumstances of a heat wave, **however, failure to constructively challenge this independence could conceivably contribute to the vulnerability of elderly people**

One social contact was worried about their relative’s illness which was exacerbated by the heat, and had in the past acted on it. Another social contact was concerned about heat risks because ofthe direct experience ofthe elderly having suffered from heat stroke some years ago.

The dominant, although not uniform, perception among social contacts of the elderly seems to be one of resilience and common sense compromised only by serious illness, reduced mobility and/ or disability. Evidence for this is well demonstrated by the exemplary quote ‘‘She’s quite sensible regarding the heat!’’ (Norwich primary respondent, female, age 57, about her 80year-old parent). The quote encapsulates the notion that most elderly people, as long as they are sensible, cope well with heat.

In either case, among both peers and younger social contacts, the primary narrative describes capable, competent and independent elderly people who are well equipped to take care of themselves in the face ofheat risk. Social contacts did not generally challenge the perceptions of their relatives or friends relating to resilience and independence.

As a result, bonding networks act as vehicles of narratives that uphold the lack of prevention and legitimise the reactive approach so prevalent among elderly responses to heat waves. The narratives are founded in perceptions that do not regard heat as a signiﬁcant threat and are constructed by passing information which supports reactive strategies to heat. **Combinedwith a knowledge deﬁcit, and attitudes that encourage independence, these narratives could heighten vulnerability and potentially prevent anticipatory adaptation to the effects of heat waves**

In particular, those networks that lack vertical linkages and linkages to people on the outside ofthe network, whomay not share similar social identities, have been pointed to as sources of potential conﬂict, albeit this may not be a robust ﬁnding to date (cf. Szreter and Woolcock, 2004).

The bonding networks evidenced in this research probably exist in a context of other types of networks. Individuals interviewed here may engage in other types of social capital which could potentially counteract the narratives transmitted through bonding networks.

The evidence presented here points to a less than straightfor- ward relationship between social capital, vulnerability reduction and increasing resilience. Bonding social capital, depending on context, could play a particular role in shaping vulnerability, potentially even compromising adaptation. **But theoretically it seems that this effect could be counteracted by stronger linking and bridging social capital**. Bridging ties to distinct groups, with different values and perspectives, and linking ties upward through authority gradients could perhaps challenge the views perpetuated by bonding networks.

(Wolf et al., 2010)

Attracting under-represented groups requires more than simply physical changes to the environment. Supported activities are crucial [31]. Projects that combine technical/infrastructural approaches with education, training, and community-based interventions, are more likely to have a more profound and lasting eﬀect on behavior [12]. For example, Hunter et al. found that interventions where physical activity programs are combined with a physical change to the built environment (e.g., restructuring green space) are likely to have the largest eﬀect on physical activity [40].

(Kruizse et al., 2019)

Several studies have been focusing on heat perception and coping strategies [37–40]. Few studies are focusing on the vulnerable group of elderly people [41–43]. These studies identiﬁed a variety of coping strategies during heatwaves. Banwell et al. [41] found that the participants reported a number of coping strategies including a universal use of air conditioning, adaptation of daily activity and changing dietary habits. The use of cooling appliances as a main strategy is underlined by the ﬁndings of Kondo et al. [42] and Nitschke et al. [43], in which the efﬁcacy of targeted information was explored. They found an **increased use of cooling systems after receiving information leaﬂets**. Research in Germany on perception and coping strategies of an elderly population during episodes of heat is rare. There are only two studies focusing on heat-related behavior in older adults during heatwaves [44,45]: Lindemann et al. [44], examining a sample from sheltered care facilities, found that social participation decreases with increasing temperature whereas water intake increases; Conrad and Penger [45] showed the decreased mobility behavior during cold and hot episodes and presented a relation between health status and heat perception.

Some strategies, e.g., water-related strategies such as cooling arms and feet with water or the use of wet towels are rarely used. A review showed that taking extra showers or baths was associated with a lower risk of death during a heatwave [32]. We assume based on underuse of these diverse strategies that the participants might not know about the positive effects of these strategies, and we clearly recommend public information about the positive outcomes. In France, a survey showed that the awareness of heat risk after heat alerts broadcast on radio and television was highly associated with an increase of coping strategies (e.g., hydration, closing sun-facing windows) from 6–15% [58].

(Kemen et al., 2021)

However, in China’s rural areas, where resources have been limited and resource competitions have been high, individuals may only benefit from social capital if they are able to access certain material resources that are essential to their health, such as health care, education, nutrition, and antipollution projects (Dorsten & Li, 2010).

Thus, rural people might not be able to benefit from their family members, friends, and people like them, who are also resource deprived. It is arguable that power-related linking social capital may be the only way in rural China to bring in needed resources; however, we cannot overestimate the effect of this type of social resource, given the country’s significant widespread poverty in its prereform era and long-lasting poverty in its rural areas even after reforms. In the Chinese context, **social capital seems only to become resourceful when material resources are accessible and thus connect to beneficial health effects**. Our results among urban elders—linking social capital was associated with physical health but not emotional health—also allude to this implication. Because material resources are more critical to physical health than to emotional health, linking social capital’s beneficial health effects may only apply to elite older people who would be able to bring in resources through their connectedness with the power

(Norstrand & Xu, 2012)

**Social capital and belief in collective efficacy can increase individual self efficacy**

Collective action can have a direct influence on resource allocation in neighborhoods. Community members can increase control over their lives and environment through collective actions, which in addition to providing access to resources, may increase the capability of communities and individuals to change health-related behaviors.

Critical for the effectiveness of any collective, however, is ‘a group’s shared belief in its conjoint capabilities to organise and execute the courses of action required to produce given levels of attainment’ (Bandura 1997, 477), termed ‘collective efficacy’ in Bandura’s influential work on the exercise of control. Individuals’ belief in a group’s collective efficacy, Bandura argues, does not only affect the performance of a group but also how individuals themselves manage their own resources, strategies and motivations. This bridging characteristic of collective efficacy is supported by previous studies in non-disaster contexts, demonstrating that beliefs about the ability of a group to achieve particular goals also influence perceptions and behaviours at the individual level (e.g. Jugert et al. 2016; Ferguson and Mindel 2007). Consequently, collective efficacy may also affect attitudes and behaviours with respect to preparing for, reacting to or recovering from natural disasters.

Collective efficacy is rooted in social cognitive theory, suggesting that the key to human agency is the belief in one’s own or group capability ‘to organize and execute the courses of action required to manage prospective situations’ and that such beliefs ‘influence how people think, feel, motivate themselves, and act’ (Bandura 1995, 2). Efficacy beliefs enable human agency through four major processes, including cognitive (e.g. analytic thinking), motivational (e.g. beliefs about what can be achieved), affective (e.g. distress or anxiety) and selection processes (e.g. choosing courses of action). These efficacy-activated processes are assumed to operate in concert, ultimately regulating human functioning (Bandura 1995)

Efficacy at the individual and the group level influence each other (Bandura 1995, 1997). Bandura uses the metaphor of a sports team, where an individual team member judges her own capabilities (i.e. self-efficacy) with reference to the capabilities of the team as a whole (i.e. collective efficacy) and, vice versa, judges the team’s chances of success in the light of her own potential contribution to its success. This relationship between collective efficacy and self-efficacy has been confirmed empirically, for instance in the education context (Stephanou, Gkavras, and Doulkeridou 2013; Caprara et al. 2003; Goddard and Goddard 2001) and for pro-environmental behaviours (Jugert et al. 2016).

Risk perception, fear and self-efficacy are key explanatory factors for protective behaviour of individuals according to Protection Motivation Theory (PMT; Rogers 1975, 1983). In the PMT, risk perception is conceptualised as a combination of the perceived severity and consequences of a threat. Self-efficacy is a central component of coping appraisal and is defined as a person’s perceived ability to carry out a specific action aimed at reducing potential negative consequences (Floyd et al. 2000).

Risk perception, fear and self-efficacy are linked to the social environment: Elliott and Pais (2006, 300) emphasise that ‘people respond to disasters not as isolated individuals but as members of overlapping forms of social affiliation’. Social capital dampens risk perception and fear but increases self-efficacy of households at risk from natural hazards (Wolf et al. 2010; Babcicky and Seebauer 2017).

Socio-demographic variables, such as gender (Babcicky and Seebauer 2017; Kellens et al. 2011), age (Thistlethwaite et al. 2018; Kellens et al. 2011; Zaalberg et al. 2009) and income (Babcicky and Seebauer 2017) have a significant influence on risk perception, fear and self-efficacy.

What is striking is that these collective efficacy components have differing impacts on risk and coping beliefs: risk perception and fear are lowered by social cohesion, but are increased by efficacy belief in citizen groups engaged in preventive participatory action. Social cohesion and group efficacy do not increase self-efficacy, but **belief** in social support does.

Residents with high levels of trust in their community perceive themselves to be at lower risk and therefore may have weaker intentions to prepare for a natural disaster. In a situation of high objective disaster risk, perceiving low risk may lead to a false sense of safety among residents, as discussed in Babcicky and Seebauer (2017). To prevent such an undesired consequence, social cohesion should instead be channelled into hazard-specific collective actions, since the task-specific components of collective efficacy do not seem to bear the risk of counterproductive effects. Belief in social support and in effective citizen group action has the potential to empower residents by making them feel more capable of protecting themselves against natural hazards. If residents believe that they can tackle local risk issues effectively by joining preventive or participatory citizen groups, they tend to be more aware about natural hazards. Here, collective efficacy beliefs may spill over to the individual level, encouraging as well as empowering residents to take risk-reducing actions. Social support and group efficacy together may increase risk perception as well as self-efficacy; this combination is regarded as a pre-condition for protective behaviour (Grothmann and Reusswig 2006).

(Babcicky & Seebauer, 2020)

An attentive review of ordinary days (i.e., days devoid of major negative events or exacerbations of ongoing strains) would probably reveal that we routinely obtain demonstrations of love, caring, and understanding from intimates when we recount the day’s minor uplifts and hassles to one another. **These demonstrations can sustain a sense that we matter to those we see as significant others and undergird our self-esteem**. We also frequently swap useful information or advice with spouses, neighbors, coworkers, church members, and others as we chat about vexing circumstances encountered in our daily activities (e.g., inconvenient bank hours, mistimed traffic lights, overly expensive goods and services). Facts or recommendations from others enable subsequent behavioral changes that make everyday tasks more efficient, economical, or successful and probably **sustain our sense of mastery or control over life.** Even more importantly, informal discussions with others about impending problems at home, at work, or in other role domains may pinpoint ways to prevent the occurrence of a stressor or de-escalate the appraisal of imminent problems from possible threats to manageable challenges (Cohen and McKay 1984; S. E. Taylor and Aspinwall 1996; Thoits 1985; Uchino 2004).

(Thoits, 2011)

**Individual characteristics of elderly are important: literacy, mental and physical health**

Baker et al.’s study [77] into health literacy and mortality in older people found that poor health literacy is correlated with poor uptake of preventative services, with an overall strongest correlation between reading ﬂuency and all-cause mortality. Geboers et al.’s study [78] also found the same correlation between low education level and low health literacy. Moving beyond just reading or numeracy skills, Serper et al. [79] noted that for older people, cognitive processing such as memory, processing speed, and inductive reasoning all inﬂuence functional health. This could perhaps explain the low rates of heat warnings recall, confusion, and ignorance regarding risks to health and lack of translation to protective behaviours [47,55,57]. Another interesting point to note is the success of Nitschke et al.’s randomised controlled trial in which multiple ‘reminders’, such as laminated cards and fridge magnets, were used to facilitate adaptive behaviours [49]

(Vu et al., 2019)

high social participation was strongly associated with decreased risk of heat-related EDP and health events. This is consistent with previous studies where increased social contact has been associated with a decreased risk of heat-related mortality12 whereas stopping usual activities during a heat wave has been associated with an increased risk of heat-related morbidity.17 ADL limitation was an independent risk factor of both outcomes. **Loss of autonomy in ADL** has been previously associated with heatrelated mortality.13 Although disability and social participation may be correlated, we observed that they made distinct contributions to HRHO. Needing help in ADL signals an inability to care for oneself, which could lead to difficulties in achieving appropriate actions in order to prevent HRHO. In contrast, **high social participation may protect against extreme heat as it may increase awareness among friends/family that a person feels ill, and may provide opportunities to seek advice**.2 Promoting social participation among older adults could be an effective strategy to prevent HRHO. Age-friendly cities, an approach that supports the creation of active living communities and fosters community engagement,28 could help reach out to isolated older people, who are highly vulnerable to heat owing to a poor health status or potentially unhealthy behaviours contributing to dehydration and impaired judgement. **Low household income** was associated with increased risk of both outcomes, which is consistent with results from a study conducted during the 1999 Chicago heat wave.13 **Low income is associated with financial barriers that restrict a person’s ability to take actions to reduce his or her heat vulnerability (e.g., purchase of air conditioning)**.2

(Laverdière et al., 2016)

Residents in single family homes reported more use of ‘taking a shower’, and ‘changing clothes’ than any other residence type. In a high rise, the use of ‘opening windows or doors’, ‘turning on fans’, ‘turning on the air conditioner’, and ‘leaving the house’ had reportedly higher use than the other residence types

Some behaviors seemed to be more motivated by outdoor temperatures versus indoor temperatures. For example, the behavior of leaving the house, based on outdoor temperature, steadily increased over the pre-determined temperature intervals. This suggests that the perception of the weather being hotter – e.g., based on media reports – could encourage a person to leave the house, more so than the actual temperature indoors.

The odds of opening windows or doors, using a fan, or going to the basement were not signiﬁcantly associated with outdoor temperature which could indicate that those behaviors are more driven by indoor temperatures than outdoor temperature. The temperature a person is directly experiencing might cause them to engage in the simple behaviors that could bring some relief; such as using basements, which in most homes, are cooler than upper ﬂoors, whereas the perception of being hotter might inﬂuence them to engage in more complex behaviors. Given the relatively low prevalence of reported behavior use in this study, we suspect seniors are underutilizing the full range of heat adaptation measures. Furthermore, even though we observed a limited amount of time periods when the indoor temperature exceeded 29.4 ◦C, our data suggests that seniors may also underuse the full range of adaptive behaviors during heat waves

(White-Newsome et al., 2011)

1. **CMOC 8**

**Populations may influence provision of community oriented healtcare and social services and infrastructure**

Populations that are more engaged with civic affairs also may inﬂuence health care and social services organizations, making these services more responsive to community needs and thus better able to prevent hospital readmissions. Prior research has demonstrated that social capital inﬂuences the ability of community representatives on hospital governing boards to inﬂuence provision of communityoriented services,31 and that social capital also inﬂuences integration of social services with health care.32

(Brewster et al., 2019)

Kawachiand colleagues (23) note that a cohesive neighborhood is moresuccessful in uniting for the best interest of the neighborhood. Consequently, communities rich in social capital can be more successful in influencing political decisions and fighting cuts to local services such as health care.

Collective action can have a direct influence on resource allocation in neighborhoods. Community members can increase control over their lives and environment through collective actions, which in addition to providing access to resources, may increase the capability of communities and individuals to change health-related behaviors.

Our case community was selected on the basis of a recent experience with a successful community action process. Due to a decreasing population, the primary health care center was closed. This political decision was strongly opposed by the community and triggered several community actions. The end result was the establishment of an association-driven health center. Existing social capital in this community was characterized by high levels of civic engagement that seemed to be inherited from one generation to the next (14). **Strong and dense associations played an important role in getting people involved, and powerful ‘helping-out norms’ obligated people to engage in the community**. Strong leaders set the norms and functioned as role models for participation. Effective information channels, e.g. faceto-face meetings, guaranteed that almost everyone was invited to participate. However, those who did not engage were seen as outsiders. According toWakefield and Poland (51), strong community connections may also lead to increased social exclusion, an idea that was confirmed in our case study

(Eriksson, 2011)

**The availability of resources is important**

However, in China’s rural areas, where resources have been limited and resource competitions have been high, individuals may only benefit from social capital if they are able to access certain material resources that are essential to their health, such as health care, education, nutrition, and antipollution projects (Dorsten & Li, 2010).

Thus, rural people might not be able to benefit from their family members, friends, and people like them, who are also resource deprived. It is arguable that power-related linking social capital may be the only way in rural China to bring in needed resources; however, we cannot overestimate the effect of this type of social resource, given the country’s significant widespread poverty in its prereform era and long-lasting poverty in its rural areas even after reforms. In the Chinese context, social capital seems only to become resourceful when material resources are accessible and thus connect to beneficial health effects. Our results among urban elders—linking social capital was associated with physical health but not emotional health—also allude to this implication. Because material resources are more critical to physical health than to emotional health, linking social capital’s beneficial health effects may only apply to elite older people who would be able to bring in resources through their connectedness with the power

(Norstrand & Xu, 2012)

Strong leaders set the norms and functioned as role models for participation

(Eriksson, 2011)

**Beware of exclusion**

However, those who did not engage were seen as outsiders. According toWakefield and Poland (51), strong community connections may also lead to increased social exclusion, an idea that was confirmed in our case study

(Eriksson, 2011)

In addition, minority groups within older people, such as older migrants, were found to be reluctant to engage in participation processes. Here, disadvantaging age-related factors might intersect with migration-related factors, such as insuﬃcient language skills (Low, 2013).

In addition, some scholars have concerns that procedural injustices are often disguised as procedural justice by focusing on a “greater good”, such as the aim of implementing sustainability or climate change action plans and strategies to improve the overall environmental condition of a neighbourhood or city district. This process can go against the needs and interests of the very local population, with the tacit intention to tame, co-opt or silence their voices while beneﬁting higher income groups or developers and even to evict lower status groups or vulnerable population groups. These processes have been discussed, e.g., as green gentriﬁcation, environmental or eco-gentriﬁcation (Anguelovski et al., 2016; Checker, 2011; Dooling, 2009). In the context of older people in urban areas where there already exist barriers such as lower participation among older people and migrants in public participation, planning and politics must carefully pay attention to making the voices of all aﬀected people heard, not just those of the well-educated and well-connected who know how to set their interests on the political agenda (Novy and Colomb, 2013).

(Enssle & Kabisch, 2020)

**A shared belief in efficacy**

Critical for the effectiveness of any collective, however, is ‘a group’s shared belief in its conjoint capabilities to organise and execute the courses of action required to produce given levels of attainment’ (Bandura 1997, 477), termed ‘collective efficacy’ in Bandura’s influential work on the exercise of control. Individuals’ belief in a group’s collective efficacy, Bandura argues, does not only affect the performance of a group but also how individuals themselves manage their own resources, strategies and motivations. This bridging characteristic of collective efficacy is supported by previous studies in non-disaster contexts, demonstrating that beliefs about the ability of a group to achieve particular goals also influence perceptions and behaviours at the individual level (e.g. Jugert et al. 2016; Ferguson and Mindel 2007). Consequently, collective efficacy may also affect attitudes and behaviours with respect to preparing for, reacting to or recovering from natural disasters.

Collective efficacy is rooted in social cognitive theory, suggesting that the key to human agency is the belief in one’s own or group capability ‘to organize and execute the courses of action required to manage prospective situations’ and that such beliefs ‘influence how people think, feel, motivate themselves, and act’ (Bandura 1995, 2). Efficacy beliefs enable human agency through four major processes, including cognitive (e.g. analytic thinking), motivational (e.g. beliefs about what can be achieved), affective (e.g. distress or anxiety) and selection processes JOURNAL OF RISK RESEARCH 697 (e.g. choosing courses of action). These efficacy-activated processes are assumed to operate in concert, ultimately regulating human functioning (Bandura 1995).

Collective efficacy is closely linked to and in part overlaps with other social mechanisms, par- ticularly with social capital (Lochner, Kawachi, and Kennedy 1999). In its broadest sense, social capital refers to the ‘features of social organisation, such as trust, norms, and networks, that can improve the efficiency of society by facilitating coordinated actions’ (Putnam, Leonardi, and Nanetti 1993, 167). Despite their similarity in describing the social background against which collective action emerges, there are nuanced but important differences between the two concepts. While social capital underscores the general potential for mutual support rooted in social relationships and networks, the emphasis of collective efficacy lies on the ability of a group to leverage this potential for pursuing specific tasks (Drakulich 2014). Alternatively, as Cagney and Wen (2008, 242) succinctly put it: ‘social capital is about relationships and collective efficacy is about converting those relationships into action’; however, collective efficacy also acknowledges that collective action is embedded in the general social context and therefore consists of both a social cohesion component and a component that captures expectations about task-specific actions (Sherrieb, Norris, and Galea 2010; Ansari 2013; Sampson 2006).

In the literature on collective efficacy, the social cohesion component typically captures aspects related to mutual trust (e.g. ‘people in this neighbourhood can be trusted’) and solidarity (e.g. ‘people around here are willing to help their neighbours’) (Browning and Cagney 2002, 389). Far less attention is directed towards social networks, because the intention to act collectively is assumed to be primarily influenced by the sense of attachment to a community, rather than by the density of pre-existing social networks (Browning and Cagney 2002; Sampson 2006). The conceptualisation of the task-specific component of collective efficacy hinges on shared beliefs, either about performing particular actions or about achieving particular outcomes. Sampson, Morenoff, and Earls (1999) for instance, assess shared belief and mutual engagement regarding specific actions in child social control. Goddard (2002) and Van Zomeren, Postmes, and Spears (2008), on the other hand, stress that people’s expectations focus on whether collective action would achieve its desired outcomes effectively. Taken together, the task-specific component of collective efficacy captures a group’s conjoint capability to perform specific tasks aimed at achieving a particular goal (Zaccaro et al. 1995).

We understand collective efficacy to consist of two distinct components: social cohesion and task-specific efficacy beliefs. While social cohesion is a context-independent general quality of a community and does not refer to a particular field of action, task-specific efficacy beliefs are situational and therefore need to be tailored to a specific context (here, natural hazards) (Sampson 2006).

From a collective efficacy perspective, the general availability of social resources—commonly referred to as social capital—does not suffice to promote individual risk reduction; in addition, to activate these resources, it also requires perceived joint ability to carry out collective action. Consequently, collective efficacy unites two components: social cohesion (i.e. trust and solidarity) as the foundation for collective action, and efficacy beliefs relating to specific tasks where social resources are converted into action.

In this study, we conceptualised social cohesion as a component of collective efficacy vis-~a-vis beliefs specific to a particular task. An alternative approach would be to consider social cohesion an antecedent of the task-specific component; this seems plausible against the view that social cohesion is a resource potential and collective efficacy is the ability to draw on this resource (Drakulich 2014; Zaccaro et al. 1995). Methodologically, this translates into a causal chain from social cohesion to task-specific efficacy beliefs to risk and coping beliefs. This alternative approach has been used in the context of informal social control (Collins, Neal, and Neal 2017; Gau 2014) and merits investigation in the natural hazards context; an empirical confirmation of the assumed causal chain requires longitudinal or experimental data, though.

(Babcicky & Seebauer, 2020)

**By promoting itself a group can increase its beliefs in collective efficacy**

Collective efficacy emerges not just from the way a group is perceived by its (potential) members but may also be purposefully directed by the way the group presents itself in public discourse. To instil belief in its capabilities and to attract additional members, a citizen group might actively market its mission and successes, in a similar way to the public relations activities that businesses undertake in the commercial sector. However, the marketed objectives a group claims to be able to achieve need to be realistic; otherwise, the group’s efficacy could be questioned, or the group may even be distrusted by the community. Citizen groups could, therefore, be advised to appoint a group head or spokesperson who is experienced in communicating with different audiences through various media, such as speaking at public meetings, writing newspaper articles or participating in (online) debates.

(Babcicky & Seebauer, 2020)

**Social capital influences self-efficacy, a common goals is important**

Bonding social capital is created internally through capacity building, which serves as the motor for collective action (also some- times referred to as network power, see Booher and Innes 2002). In such cases, bonding social capital works primarily via a ‘soft infrastructure’ (Ostrom 2000 as cited in Rydin 2014), which enables the sharing of knowledge to enable under- standing and generate rules or expectations about acting collectively (Rydin 2014). We identify two dimensions of bonding social capital for the empirical study of the internal organization of activist groups faced with contestation: the construction of a common identity and purpose, and the maintenance of

First, individuals are strongly linked through a common identity and purpose. When a shared purpose or meaning for action is established, the strong ties made possible by bonding social capital maintain and reinforce this common goal. For activist communities, the reliance of one individual on the others is crucial. Without a shared reason for action, individuals may not per- ceive the need to come together, especially in difficult moments, or to share resources and knowledge (Innes and Booher 2010).

This can have a serious negative effect on the maintenance of group integrity, the second dimension, which is primarily main- tained through a process of social monitoring. This particular capacity is built through transmission of common understanding and through the ‘sanctioning of behaviors that are not in accordance with pre-established norms of the community by shaming and blaming’ (Rydin 2014, 29). Group integrity is a fundamental aspect in the long-term durability of a community, where the characteristic informality has the potential to break down internal structure. However, it is possible to develop feelings of fear, mistrust and hate towards others not in compliance with the group ideals. This alludes to the ‘dark side’ (Rydin 2014, 13) of bonding social capital and a potential side effect of community relations, exclusivity.

Bridging social capital, or the capacity of a certain group to connect with others, provides an explanation for how internal organization facilitates connections with subjects and agents outside of a core community. Such external relationships have the tendency to be less stable and are not based on long- term face-to-face forms of social interaction as in bonding social capital. We identify three main resources that might increase the bridging social capital relevant for an activist group to externally connect: the presence of intermediaries, expert advice and social media.

Bonding social capital, as we show, was weakened by fundamental discussions regarding the overall purpose of the group. Pragmatic views confronted those with a more idealistic leaning, leading to dis- cussions that ultimately disrupted the common purpose. Combined with a rel tively high degree of inclusiveness towards new members and political ideas, the group’s central purpose often fluctuated, resulting in the absence of a cohesive stance towards institutional policies proposing eviction.

﻿Maintenance of group integrity was thus dependent upon the degree (or lack thereof) of exclusiveness that the collective put into place and was most fragile during the months leading up to the eviction from the Passeerdersgracht location.

﻿This common purpose was continually strengthened, confirmed and repeated in visual, spoken and non-spoken form. Although the Undercroft skateboar- ders were characterized by informality, the common goal acted as a safeguard for con- tinuous constructive interaction, especially in the face of unexpected turns during the campaign. The Undercroft was able to reduce internal complexity, redundancy and noise, while building structure on the basis of a common, clear and un-discussed purpose. The precise goal of saving the space for skateboarding allowed the maintenance of momentum and focus, even in the midst of an otherwise spontaneous and self-regulated method of organization.

﻿When a common purpose is not defined, internalized and resistant to critique, activist groups might be more vulnerable to policy reform, policing or negotiations with authorities, mostly due to the fact that examples of urban activism are particular, very localized and often spontaneous form of civic activism.

(VanHoose & Savini, 2017)

Adlakha, D., Chandra, M., Krishna, M., Smith, L., & Tully, M. A. (2021). Designing age-friendly communities: Exploring qualitative perspectives on urban green spaces and ageing in two indian megacities. *International Journal of Environmental Research and Public Health*, *18*(4), 1–13. https://doi.org/10.3390/ijerph18041491

Alambo, F. I., & Yimam, H. A. (2019). Elderly care and social support systems among the Gedeo of southern Ethiopia: Socio-cultural contexts, forms, dynamics and challenges. *African Studies Quarterly*, *18*(3), 15–28.

Arnberger, A., Allex, B., Eder, R., Ebenberger, M., Wanka, A., Kolland, F., Wallner, P., & Hutter, H. P. (2017). Elderly resident’s uses of and preferences for urban green spaces during heat periods. *Urban Forestry and Urban Greening*, *21*, 102–115. https://doi.org/10.1016/j.ufug.2016.11.012

Babcicky, P., & Seebauer, S. (2020). Collective efficacy and natural hazards: differing roles of social cohesion and task-specific efficacy in shaping risk and coping beliefs. *Journal of Risk Research*, *23*(6), 695–712. https://doi.org/10.1080/13669877.2019.1628096

Barigozzi, F., Cremer, H., & Roeder, K. (2020). Caregivers in the family: Daughters, sons and social norms. *European Economic Review*, *130*, 103589. https://doi.org/10.1016/j.euroecorev.2020.103589

Brewster, A. L., Lee, S., Curry, L. A., & Bradley, E. H. (2019). Association between Community Social Capital and Hospital Readmission Rates. *Population Health Management*, *22*(1), 40–47. https://doi.org/10.1089/pop.2018.0030

Broese van Groenou, M. I., & De Boer, A. (2016). Providing informal care in a changing society. *European Journal of Ageing*, *13*(3), 271–279. https://doi.org/10.1007/s10433-016-0370-7

Cramm, J. M., Van Dijk, H. M., & Nieboer, A. P. (2013). The importance of neighborhood social cohesion and social capital for the well being of older adults in the community. *Gerontologist*, *53*(1), 142–150. https://doi.org/10.1093/geront/gns052

de Klerk, M., de Boer, A., & Plaisier, I. (2021). Determinants of informal care-giving in various social relationships in the Netherlands. *Health and Social Care in the Community*, *29*(6), 1779–1788. https://doi.org/10.1111/hsc.13286

Enssle, F., & Kabisch, N. (2020). Urban green spaces for the social interaction, health and well-being of older people— An integrated view of urban ecosystem services and socio-environmental justice. *Environmental Science and Policy*, *109*(September 2019), 36–44. https://doi.org/10.1016/j.envsci.2020.04.008

Eriksson, M. (2011). Social capital and health--implications for health promotion. *Global Health Action*, *4*, 5611. https://doi.org/10.3402/gha.v4i0.5611

Haan, H. De. (2005). Social and Material Appropriation of Neighborhood Space : Collective Space and Resistance in a Dutch Urban Community. *October*, 1–122.

Jennings, V., & Bamkole, O. (2019). The relationship between social cohesion and urban green space: An avenue for health promotion. *International Journal of Environmental Research and Public Health*, *16*(3). https://doi.org/10.3390/ijerph16030452

Kabisch, N., Kraemer, R., Masztalerz, O., Hemmerling, J., Püffel, C., & Haase, D. (2021). Impact of summer heat on urban park visitation, perceived health and ecosystem service appreciation. *Urban Forestry and Urban Greening*, *60*(February), 127058. https://doi.org/10.1016/j.ufug.2021.127058

Kemen, J., Schäffer-Gemein, S., Grünewald, J., & Kistemann, T. (2021). Heat perception and coping strategies: A structured interview-based study of elderly people in Cologne, Germany. *International Journal of Environmental Research and Public Health*, *18*(14). https://doi.org/10.3390/ijerph18147495

Kemperman, A., & Timmermans, H. (2014). Green spaces in the direct living environment and social contacts of the aging population. *Landscape and Urban Planning*, *129*, 44–54. https://doi.org/10.1016/j.landurbplan.2014.05.003

Kemperman, A., Van Den Berg, P., Weijs-Perrée, M., & Uijtdewillegen, K. (2019). Loneliness of older adults: Social network and the living environment. *International Journal of Environmental Research and Public Health*, *16*(3). https://doi.org/10.3390/ijerph16030406

Kruizse, H., van der Vliet, N., Staatsen, B., Bell, R., Chiabai, A., Muiños, G., Higgins, S., Quiroga, S., Martinez-Juarez, P., Yngwe, M. A., Tsichlas, F., Karnaki, P., Lima, M. L., de Jalón, S. G., Khan, M., Morris, G., & Stegeman, I. (2019). Urban green space: creating a triple win for environmental sustainability, health, and health equity through behavior change. *International Journal of Environmental Research and Public Health*, *16*(22). https://doi.org/10.3390/ijerph16224403

Laverdière, É., Payette, H., Gaudreau, P., Morais, J. A., Shatenstein, B., & Généreux, M. (2016). Risk and protective factors for heat-related events among older adults of Southern Quebec (Canada): The NuAge study. *Canadian Journal of Public Health*, *107*(3), e258–e265. https://doi.org/10.17269/CJPH.107.5599

Li, H., Luo, W., Hou, Y., Xia, Y., Yao, J., Kang, N., Deng, C., Sun, H., & Chen, C. (2021). Factors Affecting Perceived Health Benefits and Use Behaviors in Urban Green Spaces During the COVID-19 Pandemic in Southern China Megacities. *Frontiers in Public Health*, *9*(October). https://doi.org/10.3389/fpubh.2021.759444

Maas, J., van Dillen, S. M. E., Verheij, R. A., & Groenewegen, P. P. (2009). Social contacts as a possible mechanism behind the relation between green space and health. *Health and Place*, *15*(2), 586–595. https://doi.org/10.1016/j.healthplace.2008.09.006

Markevych, I., Schoierer, J., Hartig, T., Chudnovsky, A., Hystad, P., Dzhambov, A. M., de Vries, S., Triguero-Mas, M., Brauer, M., Nieuwenhuijsen, M. J., Lupp, G., Richardson, E. A., Astell-Burt, T., Dimitrova, D., Feng, X., Sadeh, M., Standl, M., Heinrich, J., & Fuertes, E. (2017). Exploring pathways linking greenspace to health: Theoretical and methodological guidance. *Environmental Research*, *158*(June), 301–317. https://doi.org/10.1016/j.envres.2017.06.028

Menec, V. H., Means, R., Keating, N., Parkhurst, G., & Eales, J. (2011). Conceptualizing age-friendly communities. *Canadian Journal on Aging*, *30*(3), 479–493. https://doi.org/10.1017/S0714980811000237

Mittermüller, J., Erlwein, S., Bauer, A., Trokai, T., Duschinger, S., & Schönemann, M. (2021). Context-specific, user-centred: Designing urban green infrastructure to effectively mitigate urban density and heat stress. *Urban Planning*, *6*(4), 40–53. https://doi.org/10.17645/up.v6i4.4393

Mohnen, S. M., Völker, B., Flap, H., & Groenewegen, P. P. (2012). Health-related behavior as a mechanism behind the relationship between neighborhood social capital and individual health - A multilevel analysis. *BMC Public Health*, *12*(1). https://doi.org/10.1186/1471-2458-12-116

Neufeld, A., & Harrison, M. (1995). Reciprocity and Social Support in Caregivers’ relationships: Variations and Consequences. *Qualitative Health Research*, *5*(3).

Norstrand, J. A., & Xu, Q. (2012). Social capital and health outcomes among older adults in China: The urban-rural dimension. *Gerontologist*, *52*(3), 325–334. https://doi.org/10.1093/geront/gnr072

Peters, K., Elands, B., & Buijs, A. (2010). Social interactions in urban parks: Stimulating social cohesion? *Urban Forestry and Urban Greening*, *9*(2), 93–100. https://doi.org/10.1016/j.ufug.2009.11.003

Picascia, S., & Mitchell, R. (2022). Social integration as a determinant of inequalities in green space usage: Insights from a theoretical agent-based model. *Health & Place*, *73*(June 2021), 102729. https://doi.org/10.1016/j.healthplace.2021.102729

Ryan, R. M., & Deci, E. L. (2000). Intrinsic and Extrinsic Motivations: Classic Definitions and New Directions. *Contemporary Educational Psychology*, *25*(1), 54–67. https://doi.org/10.1006/ceps.1999.1020

Schmidt, T., Kerr, J., & Schipperijn, J. (2019). Associations between neighborhood open space features and walking and social interaction in older adults-a mixed methods study. *Geriatrics (Switzerland)*, *4*(3). https://doi.org/10.3390/geriatrics4030041

Seaman, P. J., Jones, R., & Ellaway, A. (2010). It’s not just about the park, it’s about integration too: Why people choose to use or not use urban greenspaces. *International Journal of Behavioral Nutrition and Physical Activity*, *7*, 1–9. https://doi.org/10.1186/1479-5868-7-78

Sharifi, E., & Boland, J. (2017). Heat Resilience in Public Space and Its Applications in Healthy and Low Carbon Cities. *Procedia Engineering*, *180*, 944–954. https://doi.org/10.1016/j.proeng.2017.04.254

Thoits, P. A. (2011). Mechanisms linking social ties and support to physical and mental health. *Journal of Health and Social Behavior*, *52*(2), 145–161. https://doi.org/10.1177/0022146510395592

Uehara, E. (1995). Reciprocity Reconsidered: Gouldner’s “Moral Norm of Reciprocity” and Social Support. *Journal of Social and Personal Relationships*, *12*(4).

VanHoose, K., & Savini, F. (2017). The social capital of urban activism: Practices in London and Amsterdam. *City*, *21*(3–4), 293–311. https://doi.org/10.1080/13604813.2017.1325207

Vu, A., Rutherford, S., & Phung, D. (2019). Heat health prevention measures and adaptation in older populations—a systematic review. *International Journal of Environmental Research and Public Health*, *16*(22). https://doi.org/10.3390/ijerph16224370

Wan, C., & Shen, G. Q. (2015). Encouraging the use of urban green space: The mediating role of attitude, perceived usefulness and perceived behavioural control. *Habitat International*, *50*(2010), 130–139. https://doi.org/10.1016/j.habitatint.2015.08.010

White-Newsome, J. L., Sánchez, B. N., Parker, E. A., Dvonch, J. T., Zhang, Z., & O’Neill, M. S. (2011). Assessing heat-adaptive behaviors among older, urban-dwelling adults. *Maturitas*, *70*(1), 85–91. https://doi.org/10.1016/j.maturitas.2011.06.015

Wolf, J., Adger, W. N., Lorenzoni, I., Abrahamson, V., & Raine, R. (2010). Social capital, individual responses to heat waves and climate change adaptation: An empirical study of two UK cities. *Global Environmental Change*, *20*(1), 44–52. https://doi.org/10.1016/j.gloenvcha.2009.09.004

Young, A. F., Russell, A., & Powers, J. R. (2004). The sense of belonging to a neighbourhood: Can it be measured and is it related to health and well being in older women? *Social Science and Medicine*, *59*(12), 2627–2637. https://doi.org/10.1016/j.socscimed.2004.05.001

Zhang, Y., van Dijk, T., Tang, J., & van den Berg, A. E. (2015). Green space attachment and health: A comparative study in two urban neighborhoods. *International Journal of Environmental Research and Public Health*, *12*(11), 14342–14363. https://doi.org/10.3390/ijerph121114342
